# Structural investigation, theoretical and biological studies of 8-hydroxyquinoline azo ligand and its metal chelates

**DOI:** 10.1038/s41598-026-58448-4

**Published:** 2026-06-22

**Authors:** Mohamed M. Elkhouly, Faten M. Atlam, Eman A. Bakr, Mohamed Gaber, Hoda A. El-Ghamry

**Affiliations:** Chemistry Department, Faculty of Science, Tanta University, Tanta, 31527 Egypt

**Keywords:** 8-Hydroxyquinoline azo, 4-Amino-2,6-dimethylpyrimidine, Metal complexes, Antitumor, Antimicrobial, ADMET, Cancer, Chemistry, Computational biology and bioinformatics, Drug discovery

## Abstract

In the current work, three novel bimetallic Ni(II), Cd(II), and Pt(II) complexes of the azo dye ligand with the name 5-(2,6-dimethyl-pyrimidin-4-ylazo)-quinolin-8-ol were planned as our target for synthesis and application in the medicinal field. The free ligand and the synthesized metal complexes were subjected to all the available analytical and spectral tools to get a clear and correct insight into their structures and geometries. Such tools supported the formation of bimetallic complexes with 4-coordination geometry, as assured from the results of elemental analysis, mass, infrared (IR) spectroscopy, and thermal analysis. Magnetic moment of 2.87 B.M for Ni(II) complex (per one Ni centre) along with UV-Vis spectra assured its tetrahedral geometry. Cd(II) complex was found to be tetrahedral, and Pt(II) complex is square planar. Biological evaluations demonstrated significant anticancer and antibacterial efficacy of most of the tested compounds. The highest activity as antimicrobial agent was afforded by the ligand, showing an inhibition zone diameter of 51 and 52 mm against Aspergillus fumigatus and Bacillus subtilis, respectively, showing higher activity than the metal complexes. The Cd(II) exhibited the highest efficacy, among all the tested compounds, toward both screened tumor cell lines (yielding IC_50_ of 3.44 ± 0.12 µg/ml for HepG-2 and 4.91 ± 0.26 µg/ml for the MCF-7 line). Computational analysis via Density Functional Theory (DFT) was conducted to elucidate the electronic and structural properties of the synthesized complexes. Geometric optimizations validated a square planar geometry for the Pt-complex, while the Ni and Cd-complexes exhibited distorted tetrahedral geometries. Analysis of the energy profiles, dipole moments, and the energy separation between HOMO and LUMO levels provided insight into the chemical stability and reactivity of the compounds. Furthermore, molecular electrostatic potential (MEP) mapping identified key sites for nucleophilic and electrophilic interactions. To evaluate their pharmacological viability, SwissADME analysis was employed to assess essential pharmacokinetic profiles, including lipophilicity, solubility, and drug-likeness, positioning these complexes as potential therapeutic candidates.

**Supplementary Information:**

The online version contains supplementary material available at 10.1038/s41598-026-58448-4.

## Introduction

Major global issues include the persistence of chronic inflammation and the prevalence of pathogenic diseases, which become worse by the growth of microbial pathogens exhibiting insensitivity to antibiotics^1^. The need for new antimicrobials is still significant despite progress in chemotherapy and antibiotics. Similarly, cancer is a serious health concern that is characterized by uncontrolled cell division and proliferation mechanisms. There is an urgent need for cancer medications with more efficacy, less damage to healthy cells, and clear targets for action, even if novel cancer treatments have been developed^2^. Azo dyes are basic chromophores that have many uses in industry, science, and medical applications^3,4^.

Azo-based ligands, characterized by their -N = N- linkages that, after transition metal complexes are formed, are characterized by their varied roles in biological systems and their significant pharmacological impact^5^. Many scientists and researchers have been attracted to the azo-dyes’ activity in order to synthesize more novel coordination complexes with enhanced antimicrobial characteristics^6^. The alternative reported azo-dye metal complexes demonstrate antimicrobial^7–9^, anticancer^8,10,11^, antituberculosis^12^, antioxidant^13^, anti-inflammatory^14^, antileishmanial^15^, antiviral activities^16^, and many more activities^17^. Azo-metal complexes offer benefits, including improved coloration and interactions, and recent studies indicate that cytotoxicity is decreased even at low concentrations^18^.

Heterocyclic coupling compounds, specifically nitrogen and sulfur-containing systems like pyrimidine, thiophene, and pyridine, represent essential building blocks in the design of high-performance azo-derivatives. These scaffolds, along with indole and quinolone derivatives, are also of great importance in industry and other advanced fields due to other applications^19^. Therefore, several mono-azo compounds with one or two heterocyclic rings have been synthesized and their spectroscopic features studied in recent decades^20^.

Quinoline and its derivatives, which are formed from synthetic or natural active substances, are crucial N-containing heterocyclic compounds for the synthesis of novel pharmaceuticals^21^. The 8-hydroxyquinoline scaffold represents a critical building block in drug design, reported for its versatile therapeutic applications. Its activity for metal chelation significantly enhances its efficacy in anticancer^22^, antimicrobial^23^, anti-HIV^24^, and anti-filarial^25^. Furthermore, the structural frameworks of 8-hydroxyquinoline (8-HQ) when integrated with azo-functionalized derivatives are significant for several metal ions as chelating agents in coordination chemistry^26–31^. The antibacterial action of 8-hydroxyquinoline and related derivatives is closely related to their metal-binding affinity since certain metal ions are necessary cofactors for many physiologically active enzymes. According to extensive research, its bactericidal mechanism of action depends on this chelation process to interfere with vital enzymatic pathways in pathogenic microbes^32^.

Pyrimidines, as a class of heterocyclic compounds, are crucial to many biological processes and have significant uses in a number of industries, such as materials science, medicine, and agriculture^33^. The development of azo-based pyrimidines is one of the primary uses of pyrimidines. Pyrimidine moieties can provide azo dyes with unique characteristics, including better light-fastness, enhanced solubility, and altered spectrum characteristics, making them extremely useful in a range of applications^34^. Furthermore, the presence of pyrimidine moieties may affect how the dye interacts with other electrophilic targets and may offer chances for specific binding, identification, and detection in biological systems^2^. Pyrimidine-based heterocyclic ligand metal complexes have demonstrated outstanding potential in a wide range of medicinal applications. Specifically, these coordination compounds have strong anticancer, antibacterial, and antioxidant properties in addition to their effective coordination with DNA molecules and cleavage abilities, rendering them excellent candidates for drug design^35^.

The utilization of computer tools has significantly increased during the last few decades^36^. Over time, the DFT has been the computational method that is most commonly used because of its greatest efficiency (better accuracy with lower processing costs)^37^.

Based on the previous facts demonstrating the biological importance of azo dyes incorporating quinoline moiety and their metallic derivatives, we designed our work to look for new metal complexes with promising biological importance. So in the present work, we report the successful synthesis, characterization, and computational modelling of a novel azo-chelating system, (E)-5-((2,6-dimethylpyrimidin-4-yl) diazenyl) quinolin-8-ol (**MPAQ)** and its series of divalent Ni(II), Cd(II), and Pt(II) metallic chelates. Biological activities, including antimicrobial and anticancer of the 3 synthesized chelates have been investigated and compared with those of the ligand and well-known medicines. These compounds’ structures were confirmed using a complementary set of analytical and spectroscopic techniques. Computational modeling based on Density Functional Theory (DFT) was applied to investigate the thermodynamic stability and determine the minimum energy of the metal complexes, thereby validating the experimentally confirmed structures.

## Experimental

### Reagents and equipment

All reagents, including 8-hydroxyquinoline, 4-amino-2,6-dimethyl pyrimidine, and the metal salts NiCl_6_.6H_2_O, CdCl_2_.H_2_O and K_2_PtCl_4_ were provided by Sigma-Aldrich and utilized as received without further purification. Methanol (MeOH), dimethyl formamide (DMF), and dimethyl sulfoxide (DMSO) were purchased from SDFCL and VWR chemicals. Details regarding the instrumentation used for structural characterization are provided in section S1 of the supplementary data.

### Ligand (MPAQ) synthesis method

**Fig. 1:**
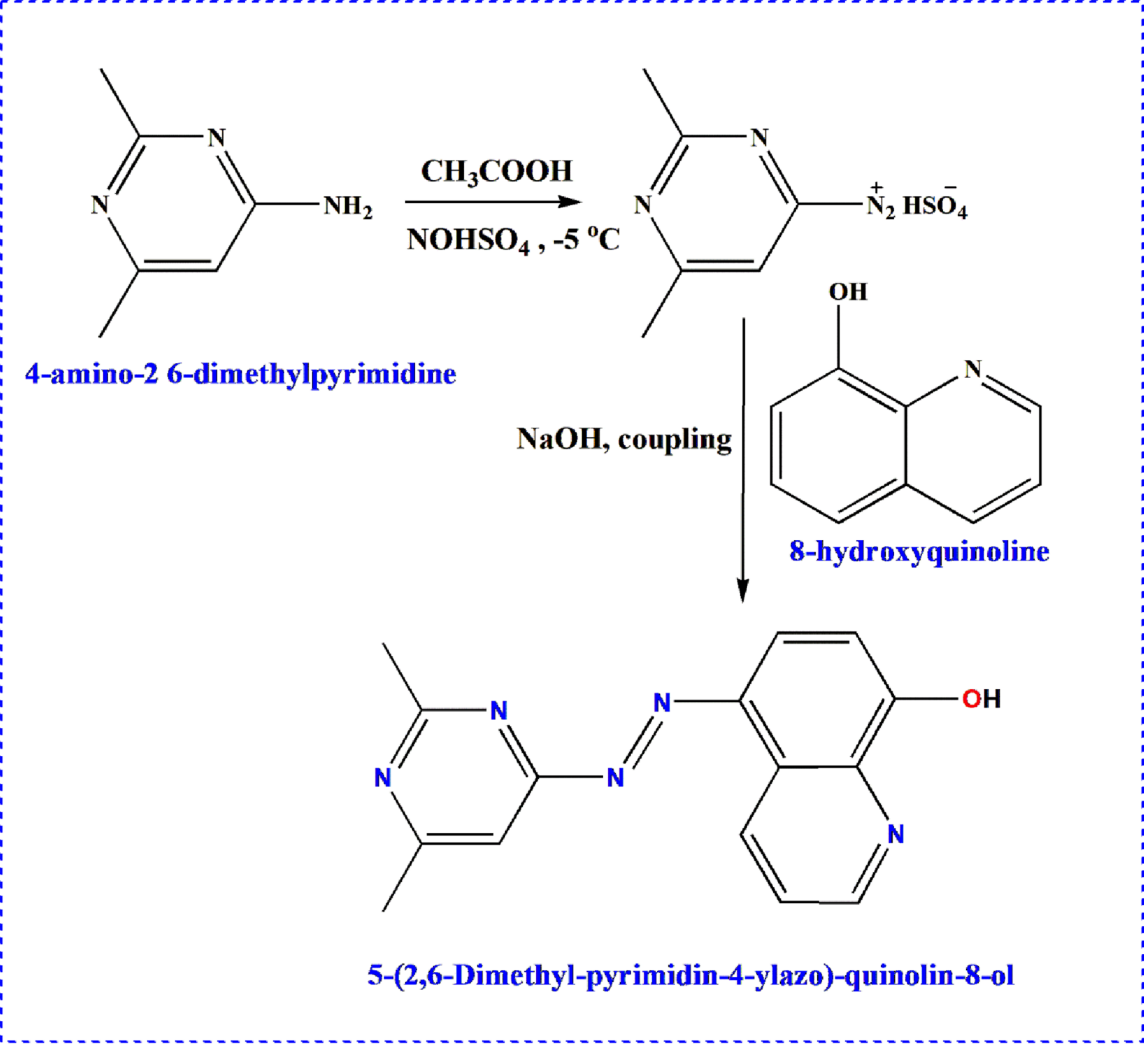
Synthesis and structure of **MPAQ** ligand along with IUPAC name.

The target azo dye was obtained by slightly altering the previously reported method^20^, which involved dissolving 4.7 mmol (0.578 g) of 4-amino-2,6-dimethylpyrimidine in 2 mL of glacial acetic acid, and the obtained mixture was then followed by 10 min of cooling in an ice-water bath. This solution was added slowly over 30 min to a precooled nitrosyl sulfuric acid solution that was prepared by slow addition of sodium nitrite (0.328 g, 4.7 mmol) to cold conc. sulfuric acid, 98% (7 mL) over 1 h. The reaction mixture was continuously stirred for an additional hour at 0 °C to form the diazonium salt. During this period, 8-hydroxyquinoline was prepared by dissolving 0.681 g (4.7 mmol) in an aqueous NaOH solution (0.188 g, 4.7 mmol NaOH). The formed diazonium salt was added slowly to the 8-hydroxyquinoline solution with vigorous stirring of the final resultant mixture. The formed deep brown material was stirred in the ice bath for 2 h, followed by keeping in the fridge overnight. The resulting precipitate was separated by filtration, washed with hot distilled water, and dried in a desiccator over anhydrous calcium chloride. It was then recrystallized from hot methanol to yield deep brown crystalline material. The purity of the product was monitored using thin-layer chromatography (TLC). Figure 1 represents the synthetic route of **MPAQ** along with its IUPAC name.

Yield: 72%; M.p.133–136 °C; Color: Deep brown; Anal. Calcd (%) for **MPAQ** (C_15_H_13_N_5_O; 279.30 g mol^− 1^): C, 64.51; H, 4.69; N, 25.07. Found: C, 64.32; H, 4.85; N, 25.31; EI-MS m/z = 279.33 [M], Calcd: 279.11. IR (wavenumbers, cm^− 1^): 3391 (υ(hydroxyl)), 1651 (υ(azomethine) pyrimidine), 1564 (υ(azomethine) quinoline), 1452 (υ(azo)), 1205 (υ(C-O)); ^1^H-NMR (DMSO-d_6_,δ, ppm): δ: 9.57 (s, 0.11 H, OH), 8.84 (d, *J* = 3.2 Hz, 1H), 8.60 (d, *J* = 6.4 Hz, 1H), 7.95 (d, *J* = 8.4 Hz, 1H), 7.70 (t, *J* = 4.8 Hz 1H), 7.44–7.36 (m, 1H), 6.69 (d, *J* = 8.4 Hz, 1H), 2.23 (s, 3 H, CH_3_), 2.12 (s, 3 H, CH_3_); UV − Vis (λ, nm): 227, 300, 340, 435.

### Metal chelates synthesis

The metal complexes of the **MPAQ** ligand were synthesized using similar procedures, as detailed below:

#### The Ni (II) complex (MPAQ-Ni)

Using 20 ml of hot ethanol solvent, 0.002 mol (0.558 g) of **MPAQ** ligand was completely dissolved and then mixed with 2 mmol of NiCl_2_.6H_2_O (0.474 g) that was previously solubilized in 10 ml of the same solvent i.e. ethanol. For about 4 h, the resulting mixture was kept for stirring and refluxing. During this time, the hopefully desired complex appeared with a colour that is different from the colours of the original reactants, upon addition of a few drops of trimethylamine (pH = 9-9.5). Filtration was used to separate the colored precipitate, and then ethanol was used for washing. The resulting precipitate was dried in a desiccator containing anhydrous CaCl_2_. Yield: 64%; M.p. Higher than 300 ^°^C. Colour: Green. Anal. Calcd (%) for **MPAQ-Ni**; [Ni_2_(MPAQ)(H_2_O)Cl_3_]⋅3H_2_O (C_15_H_20_Cl_3_N_5_Ni_2_O_5_); 574.10 g mol^− 1^: C, 31.38; H, 3.51; N, 12.20; Ni, 11.61; Found: C, 31.60; H, 3.62; N, 12.47; Ni, 11.85. Molar conductance (Λ_m_, ohm^− 1^ cm^2^ mol^− 1^, 10^− 3^ M in DMSO): 23.40. EI-MS: m/z = 574.10 [M], Calcd: 574.60. IR (wavenumbers, cm^− 1^): 3493 (υ(hydroxyl)), 1657 (υ(azomethine) Pyrimidine), 1578 (υ(azomethine) quinoline), 1463 (υ(azo)), 1233 (υ(C-O)), 504 (υ(M-O), 440 (υ(M-N)). UV − Vis (λ, nm): 270, 345, 434, 450, 540, 670, 726.

#### The Cd (II) complex (MPAQ -Cd)

Following the previously described procedure, CdCl_2_.H_2_O (2 mmol & 0.403 g) dissolved using 10 ml of hot ethanol was added as drops to 20 ml of hot ethanolic solution, within which 0.002 mol (0.558 g) **MPAQ** ligand was solubilized and stirred. after 4 h of stirring at the boiling temperature, the coloured product appeared directly. The precipitated complex was separated through filtration, washed with ice-cold ethanol, and dried. The resulting precipitate was kept in a desiccator containing anhydrous CaCl_2_ for drying.

Yield: 67%; m.p. Higher than 300 ^°^C. Colour: Light brown. Anal. Calcd (%) for **MPAQ-Cd**; [Cd_2_(MPAQ)(H_2_O)Cl_3_] (C_15_H_14_Cd_2_C_l3_N_5_O_2_); 627.48 g mol^− 1^: C, 28.71; H, 2.25; N, 11.16; Cd, 10.27; Found: C, 28.93; H, 2.46; N, 11.39; Cd, 10.69. Molar conductance (Λ_m_, ohm^− 1^ cm^2^ mol^− 1^, 10^− 3^ M in DMSO): 11.58. EI-MS: m/z = 627.48 [M], Calcd: 627.65. IR (wavenumbers, cm^− 1^): 3457 (υ(hydroxyl)), 1606 (υ(azomethine) Pyrimidine), 1577 (υ(azomethine) quinoline), 1460 (υ(azo)), 1231 (υ(C-O)), 544 (υ(M-O), 442 (υ(M-N)). UV − Vis (λ, nm): 281, 332, 342, 438, 453.

#### The Pt (II) complex (MPAQ -Pt)

2 mmol of K_2_PtCl_4_ (0.829 g) dissolved in 10 ml of hot mixed solvents (1 : 1 (H_2_O : ethanol) was added dropwise into 20 mL of a heated ethanol solution containing the ligand (2 mmol & 0.558 g). After short period of stirring and reflux, coloured material appeared in an extensive amount. The reaction mixture was heated under reflux for 2 h to ensure completion. The precipitated complex was collected and dried as previously mentioned.

Yield: 75%. M.p. More than 300 ^°^C. Colour: Faint brown. Anal. Calcd (%) for MPAQ-Pt; [Pt_2_(MPAQ)(H_2_O)Cl_3_] (C_15_H_14_Cl_3_N_5_O_2_Pt_2_); 792.82 g mol^− 1^: C, 22.72; H, 1.78; N, 8.83; Pt, 49.21; Found: C, 23.01; H, 1.94; N, 8.97. Molar conductance (Λ_m_, ohm^− 1^ cm^2^ mol^− 1^, 10^− 3^ M in DMSO): 16.73. EI-MS: m/z = 792.72 [M], Calcd: 792.82. IR (Wavenumbers, cm^− 1^): 3402 (υ(hydroxyl)), 1638 (υ(azomethine) Pyrimidine), 1576 (υ(azomethine) quinoline), 1461 (υ(azo)), 1220 (υ(C-O)), 585 (υ(M-O)), 421 (υ(M-N)). UV − Vis (λ, nm): 290, 305, 348, 440, 451, 500.

### Computational details

Using Density Functional Theory (DFT) as implemented in Gaussian 09^38^, the molecular geometries of the synthesized ligand and its Ni(II), Cd(II), and Pt(II) complexes were fully optimized without imposing any symmetry constraints. The calculations were performed employing the Becke three-parameter exchange functional in conjunction with the Lee–Yang–Parr correlation functional (B3LYP)^39^. This hybrid functional was selected owing to its well-established reliability in describing the structural and electronic properties of both organic molecules and transition-metal coordination compounds. Furthermore, B3LYP has been extensively applied in studies of metal complexes, where it provides a favorable compromise between computational efficiency and predictive accuracy, yielding geometrical parameters and electronic descriptors that are generally in good agreement with experimental observations.For the ligand atoms (H, C, N and O), the 6-311 + G(d, p) basis set was utilized^40^. This triple-zeta split-valence basis set, augmented with diffuse and polarization functions, enables an accurate representation of electron density distribution and molecular electronic properties. The metal atoms, Ni(II), Cd(II), and Pt(II), were described using the LANL2DZ basis set in conjunction with the corresponding effective core potentials (ECPs). The use of ECPs significantly reduces the computational cost while adequately accounting for the core electrons and relativistic effects that are particularly important for heavier elements such as Cd and Pt. Accordingly, the selected computational protocol has been widely employed for transition-metal complexes and provides a reliable framework for evaluating their structural and electronic characteristics^41^.

To confirm that all optimized geometries represent true local minima on the potential energy surface, harmonic vibrational frequency calculations were conducted, ensuring the absence of imaginary frequencies. The electronic characteristics of the investigated species were then evaluated via frontier molecular orbital (FMO) analysis and molecular electrostatic potential (MEP) mapping using GaussView^42^. These computational descriptors provided qualitative insights into the electronic distribution, charge localization, and primary reactive sites of both the free ligand and its corresponding metal complexes.

Quantum chemical parameters like the ionization potential (*I*) and electron affinity (*A*) can be expressed in terms of the frontier orbital energies (E_HOMO_) and (E_LUMO_) as shown in Eqs. 1 and 2:

1 $${\text{I }} = {\text{ }} - {\text{ E}}_{{{\mathrm{HOMO}}}}$$

2 $${\text{A }} = {\text{ }} - {\mathrm{E}}_{{{\mathrm{LUMO}}}}$$

The following relations were employed to calculate the chemical hardness, η, and chemical potential, µ:

3 $$\eta = {\text{ }}\left( {{\mathrm{I}} - {\mathrm{A}}} \right)/{\mathrm{2}}$$

4 $$\mu {\text{ }} = {\text{ }} - {\text{ }}\left( {{\text{I }} + {\text{ A}}} \right)/{\mathrm{2}}$$

where I and A are the first ionization potential and electron affinity of the chemical species^43^. Electronegativity can be expressed as (5):

5 $$\chi {\text{ }} = {\text{ }} - \mu$$

Parr et al.^44,45^ proposed the global electrophilicity power of a ligand as (6):

6 $$\omega {\text{ }} = {\text{ }}\mu ^{{\mathrm{2}}} /{\mathrm{2}}\eta$$

The electrophilicity index (ω) measures how much a system stabilizes its energy when it acquires more electronic charge from its surrounding environment. This descriptor takes into consideration the system’s resistance to electronic exchange as well as charge-accepting capability of an electrophile. It is a superior indicator of global chemical reactivity since it takes into consideration both chemical potential (electron transfer) and chemical hardness (stability). Conversely, chemical softness (σ), which is the reciprocal of hardness (η), measures the degree of an atom’s reactivity. The maximum electron inflow from the donor to the acceptor is calculated as follows (7):

7 $$\Delta {\mathrm{N}}_{{{\mathrm{max}}}} = {\text{ }} - {\text{ }}\mu /{\text{ }}\eta$$

### Biological studies

#### Antimicrobial activity studies

At the microbiology laboratories of Al-Azhar University, the synthesized compounds were tested for antibacterial activity using the standard disk diffusion method^46,47^. The substances’ impact as antimicrobial agents was screened using Gram-negative bacteria (*Escherichia coli* and *Proteus vulgaris*), Gram-positive bacteria (*Staphylococcus aureus* and *Bacillus subtilis*), and the fungal strains *Aspergillus fumigatus* and *Candida albicans*. Experimental details are represented in the supplementary file (section S2).

#### Antitumor activity studies

The HepG2 human liver cancer cell line and the MCF-7 human breast cancer cell line, which were obtained from the American Type Culture Collection (ATCC, Rockville, MD) were used to evaluate the compounds’ in vitro antitumor activities^48,49^; the studies were done at the Regional Centre for Mycology & Biotechnology, Al-Azhar University, Egypt. Section S3 in the supplementary file presents the detailed methodology of such a study.

### In silico studies

#### Molecular docking simulation

Molecular docking simulations were conducted using the MOE-Dock 2014 program^50^. The proteins selected for the docking investigation are the topoisomerase II in liver cancer (PDB ID: 4FM9) to assess the anticancer activity and the Bacillus subtilis protein (PDB ID: 3WHI) to assess the antibacterial activity. The docking investigations are described in Supplementary Data (section S4).

#### Drug-likeness and ADMET prediction

The SwissADME web platform was employed to evaluate ADMET profiles of the investigated compounds ( http// www.swissadme.ch)^51^.

## Results and discussion

### ^1^H-NMR spectra

**Fig. 2:**
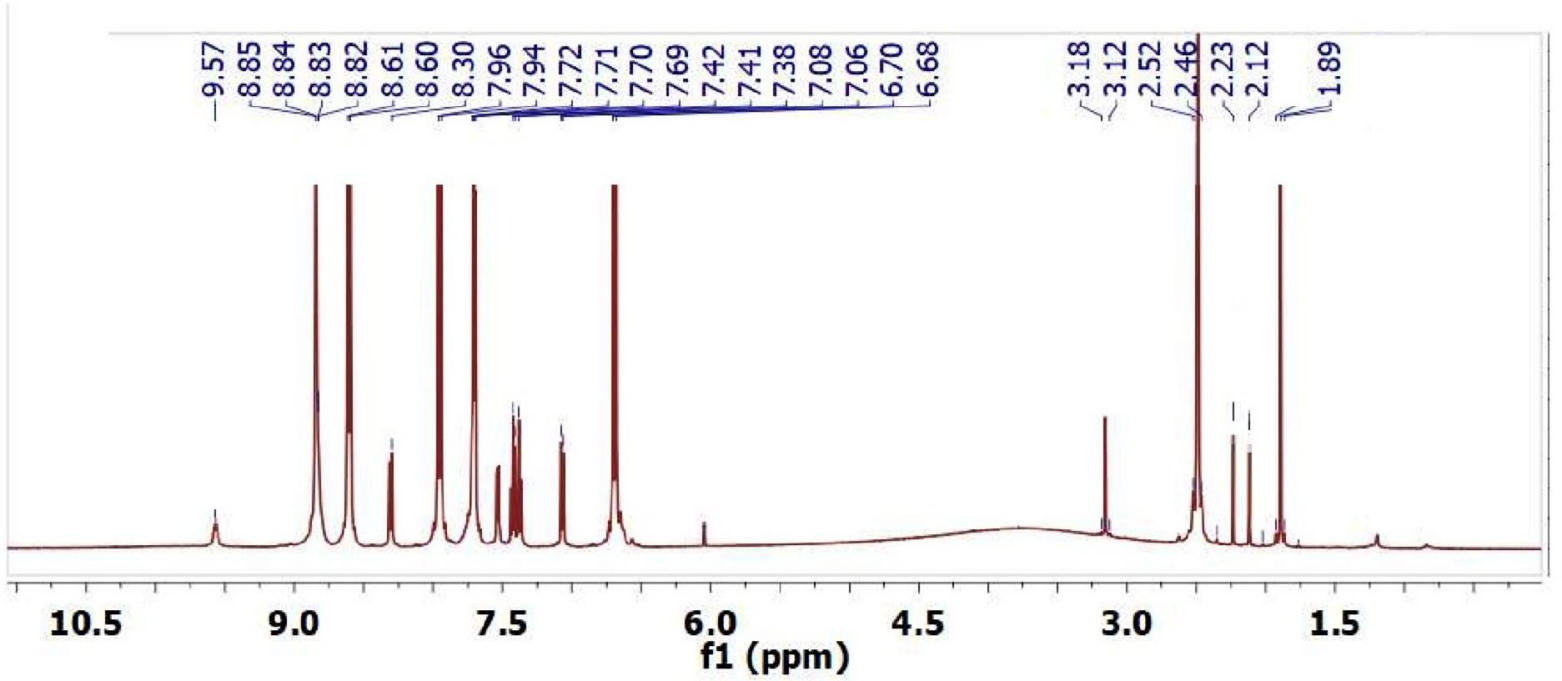
^1^H-NMR spectrum of **MPAQ** in DMSO-d_6_.

The ^1^H-NMR spectrum of the MPAQ ligand was obtained in DMSO-d_6_, utilizing tetramethylsilane (TMS) as the internal reference (Fig. 2). The ¹H NMR spectrum of the ligand showed a singlet at 9.57 ppm corresponding to the quinoline OH proton^20^. The 5 hydrogens of the quinoline ring, in addition to the pyrimidine hydrogen, appeared as doublets or triplets at 8.84, 8.6, 7.95, 7.70, 7.40, and 6.69 ppm^52,53^. The resonances for the two non-symmetrical CH_3_ groups were observed at 2.23 and 2.12 ppm as distinct singlet signals. The existence of the signals appeared at 8.30, 7.53, and 7.07 ppm is most probably due to the coexistence of azo hydrazone tautomerism^52^. The hydrazone tautomer’s distinctive NH proton signal is absent, indicating a rapid equilibrium shift in the solution state toward the azo form^52–54^.

A comparison between the ^1^H-NMR spectra of the **MPAQ-Cd** and **MPAQ-Pt** complexes with that of the **MPAQ** ligand reveals the complete disappearance of the signal at 9.56 ppm. Such behavior arises from deprotonation of the quinoline –OH group and its coordination to Cd(II) and Pt(II) ions. The other signals appearing in the 7.07–8.69 ppm and 6.97–8.91 ppm ranges in the spectra of **MPAQ-Cd** and **MPAQ-Pt** are assigned to the 6 protons of quinoline and pyrimidine rings.

### Complexes composition

The micro-analytical results of the synthesized materials (**MPAQ** ligand and **MPAQ-Ni**, **MPAQ-Cd**, & **MPAQ-Pt**) are quite close to the predicted chemical structure. The analytical data support the proposed stoichiometry of the synthesized compounds, consistent with metal complex formation in a 2:1 (M: L) molar proportion. All synthesized compounds were found to exhibit high solubility in dimethylformamide and dimethylsulfoxide, but were sparingly soluble in commonly known non-polar solvents. The molar conductivity examined in DMF solvent at a concentration of 10^− 3^ M yielded data that ranged from 11.58 to 23.4 Ω^−1^ cm^2^ mol^− 1^. The non-electrolytic behaviour of metallic compounds is supported by these results^6,7^.

### EI-Mass spectra

**Fig. 3:**
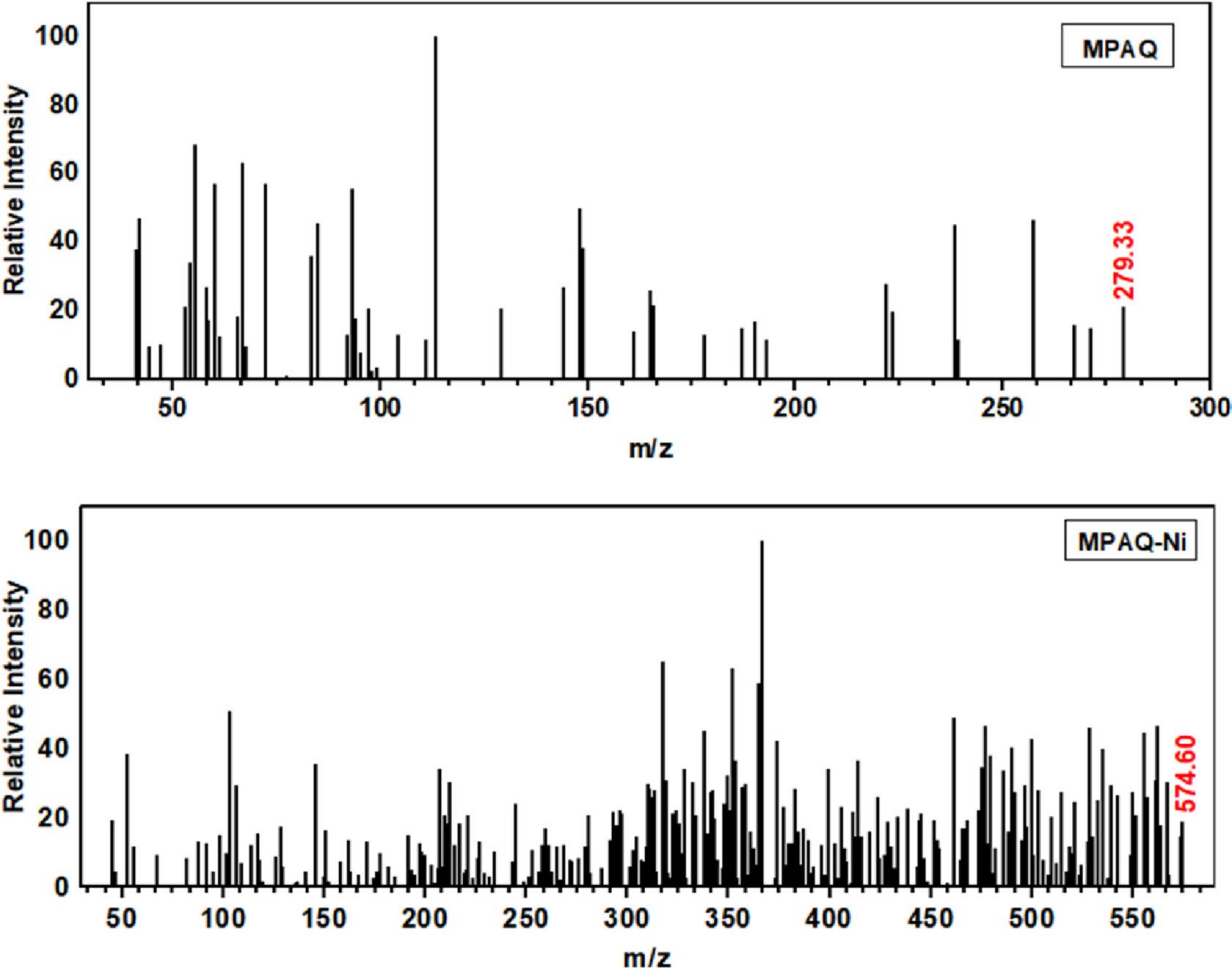
Mass spectra of **MPAQ** and **MAPQ-Ni**.

Mass spectral analysis was performed to determine the molecular weight of the **MPAQ** ligand and its corresponding metal chelates. The mass fragmentation patterns of the **MPAQ** ligand and its metal chelates are illustrated in Fig. 3 and S1, providing definitive proof of their molecular weights. Analysing these spectra indicated that the molecular ion peak of **MPAQ** emerged at m/z of 279.33, which is similar to the ligand’s molecular weight (calculated m/z = 279.30). The calculated values of 574.10, 627.48, and 792.82 correlate with the molecular ion peaks for the metal complexes, which are obvious at 574.60, 627.65, and 792.62 for **MPAQ-Ni**, **MPAQ-Cd**, and **MPAQ-Pt**, respectively. The formation of metal chelates is verified by the close agreement between the calculated and observed m/z values, which align with the empirical formulas proposed in the experimental section.

### FTIR spectra & bonding mode

**Fig. 4:**
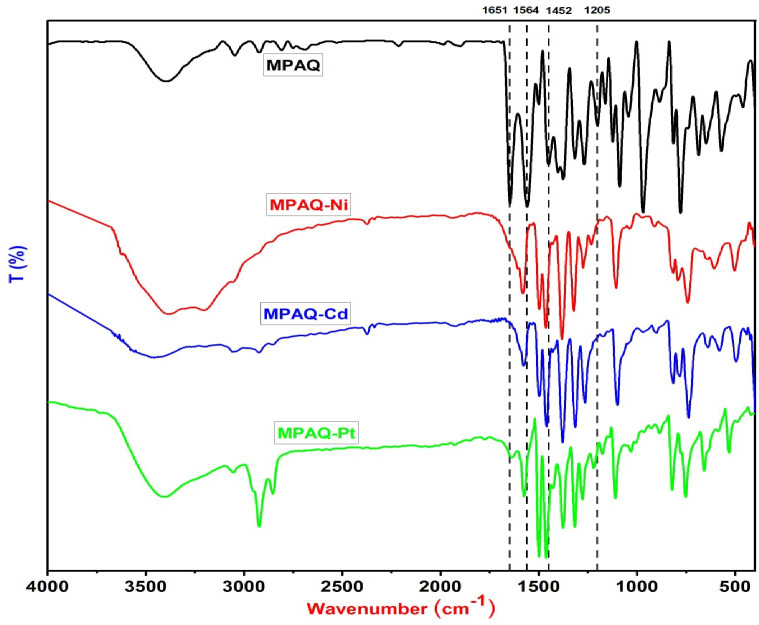
FTIR spectra of **MPAQ** and its metal chelates.

Through comparing the FTIR spectral profile of the metal complexes with that of the free ligand, the identification of the coordinating functional groups can be managed. Typically, coordinating functional groups exhibit a change in their intensity or location. Chelation may also cause certain bands to disappear. The comparative FT-IR spectra of the **MPAQ** ligand and its corresponding metal complexes are presented in Fig. 4 and the most significant infrared bands of the **MPAQ** ligand are compared to its Ni(II), Cd(II), and Pt(II) complexes. The wavenumbers of peaks are shown in the experimental section and Table S1 along with their assignments.

Analysis of the FT-IR data reveals that the broad band observed at 3391 cm^− 1^ in the spectrum of MPAQ is assigned to the stretching vibration of the hydroxyl (–OH) group. The two bands appearing at 1651 and 1564 cm^− 1^ are assigned to ν(C = N) of pyrimidine and quinoline rings, respectively. Additionally, the two peaks appearing at 1452 and 1205 cm^− 1^ are attributed to ν(N = N) and ν(C-O) groups, respectively. The absorption band corresponding to the ν(C-O) stretching vibration was observed at 1282 cm^− 1^^6,20^.

In the spectra **MPAQ-Ni**, **MPAQ-Cd**, and **MPAQ-Pt** complexes, the two bands corresponding to the ν(C = N) vibrations of pyrimidine and quinoline rings exhibited significant shifts toward either lower or higher wavenumbers, and these shifts provide strong evidence for the coordination of the ring nitrogen atoms to the metal centres. The band corresponding to ν(C = N) of pyrimidine shifted by 6–45 cm^− 1^ whereas the quinoline C = N band shifted by 12–14 cm^− 1^ as evidence of participation of C = N of both rings in coordination to the metal centres in the formed complexes^55–57^. In a similar behaviour, N = N and C-O stretching vibration bands of the ligand exhibited a remarkable shift in their position in the complexes’ spectra by 8–11 and 15–28 cm^− 1^, respectively, supporting their coordination to the metal ion centres. The appearance of a non-ligand band in the spectra of all metal complexes between 504 and 585, and 421 and 442 cm-1, which are assigned to v(M-O) and v(M-N), respectively, provides more evidence that the oxygen and nitrogen atoms are involved in complex formation.

The v(OH) stretching vibrations of water molecules present in the lattice as well as those coordinated to the metal is associated with the bands that exist in the spectra of all complexes within the range 3402–3493 cm^− 1^. The simultaneous coordination of the azomethine groups (from the pyrimidine and quinoline rings), the azo linkage, and the C–O bond supports the complexes’ synthesis in a 2:1 (M: L) molar ratio. Elemental analysis data strongly support this coordination mode.

### Thermogravimetric analytical results

**Fig. 5:**
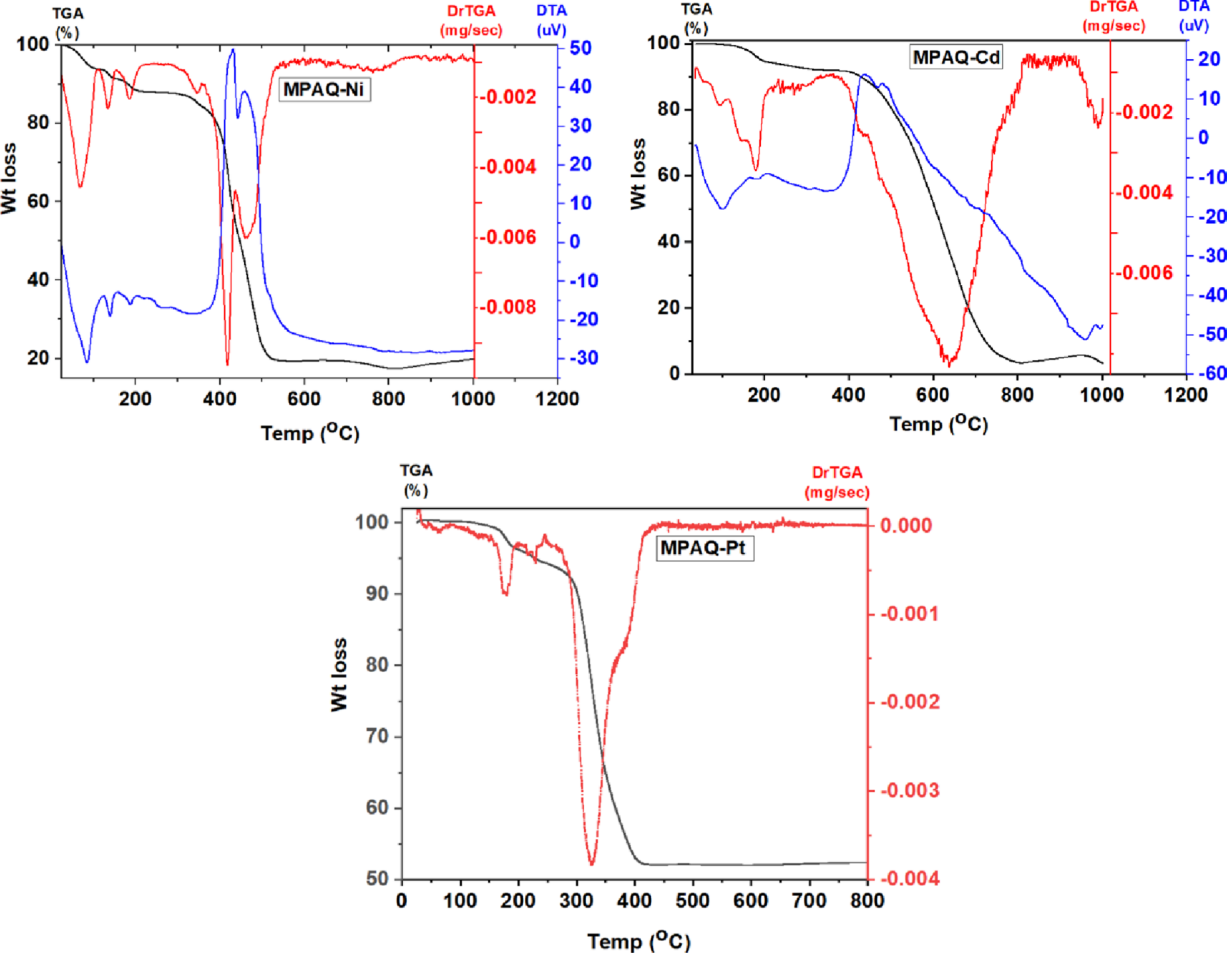
Combination of TG/DTG/DTA figures of **MPAQ-Ni**,** MPAQ-Cd** and **MPAQ-Pt** chelates.

Thermal analysis is widely recognized as an effective technique for predicting a compound’s molecular structure, it further contributes to understanding the thermal stability and decomposition pathways of the complexes, making it possible to identify the number of water molecules and the composition of the residual product. Additionally, it offers fundamental details about the compound’s thermal properties, the sequential stages of decomposition, the intermediate compounds produced, and the remaining residuals after degradation. This technique also makes it possible to detect the anionic species attached to the metal center and to determine the kind and weight% of water or organic solvents. Thermal analysis curves (TG, DTG, and DTA) for the synthesized complexes are illustrated in Fig. 5, detailing their thermal stability and decomposition profiles. Also, the analysis results are summarized in Table 1. Based on the TG curves, it is evident that **MPAQ-Cd** and **MPAQ-Pt** decomposed in three successive steps and **MPAQ-Ni** complex decomposed in five consecutive steps.

The thermal degradation of the **MPAQ-Ni** complex occurred in five distinct stages across the temperature range of 23–100, 100–145, 145–199, 199–437 and 437–512 °C. The initial step, involved the loss of two lattice water molecules corresponding to an experimental weight loss of 5.97% (calculated: 5.27%). In the second step, the last lattice water molecule was released, showing a mass loss of 3.10% (calculated: 3.13%). The third step involved the removal of one metal-coordinated water molecule, corresponding to a weight loss of 2.49% (calculated: 3.13%). These three successive steps were associated with three endothermic DTA peaks at 85, 140 and 188 °C and three peaks at 70, 134 and 186 °C in DTG curve, respectively, confirming the removal of all lattice and coordinated water from the complex. The fourth step was analysed to the loss of the chloride ions from the coordination sphere of the metal complex along with the organic fragment with the formula (C_5_H_7_N), with an observed weight loss of 33.39% (calculated: 32.65%). The DTA analysis revealed an exothermic peak at 432 °C assigned to thermal decomposition, with the DTG peak occurring at 419 °C. The loss of the remaining organic ligand was lost in the final decomposition stage, with a weight loss of 34.53% (calculated: 34.44%), resulting in the formation of Ni metal as the final residue, with a percentage of 20.52 of the total weight (calculated: 20.45%). Such a stage was accompanied by exothermic DTA and DTG peaks at 458 and 464 °C, respectively.

For **MPAQ-Cd** complex, it decomposed in three stages between 90 and 195, 195–328 and 328–800 °C. In the first stage, the simultaneous loss of one metal-coordinated water molecule together with one methyl group was recorded with a weight loss of 5.34% (calculated: 5.26%). This step is characterized by an endothermic DTA peak at 102 °C and DTG peaks at 93 and 179 °C. In the second step, the other methyl group involved in the ligand structure get lost with a weight loss of 2.56% (calculated: 2.39%). The DTG thermal peak associated with this step appeared at 279 °C. The last step showed a weight loss of 88.51%, which could not be confidently assigned due to the lack of clear identification of the resulting residues or high volatile product that is Cd metal. Consequently, no calculated value is reported for this step. The exothermic DTA peaks appeared at 434 and 482 °C and are attributed to ligand decomposition, where the DTG peak appeared at 643 °C.

Additionally, the Pt complex **MPAQ-Pt** decomposed in three stages between 155 and 192, 192–284, and 284–423 °C. In the first stage, the coordinated H₂O molecule was eliminated, accompanied by a weight loss of 3.00% of the total mass (calculated: 2.27%). In the second thermal step, the methyl group was lost with a weight loss of 2.32% (calculated: 1.89%). The third decomposition stage was attributed to the elimination of 1.5Cl_2_ that is coordinated to the metal centres, in addition to the complete decomposition of the organic ligand through the loss of the C_14_H_9_N_5_ fragment, leaving a stable residue of Pt and PtO, with observed weight loss was 43.18% (calculated: 44.60%). These TGA stages are consistent with DTG peaks at 178, 224,326, and 388 °C.

**Table 1:** Thermal results excluded from TG, DTG, and DTA thermograms of **MPAQ** and its complexes.

Complex (Mol. Wt.)	Temp. range (^°^C)	Mass loss %		DTG (^°^C)	DTA (^°^C)	Assignment
		Calcd	Found			
Ni Complex [Ni_2_(L)(H_2_O)Cl_3_]⋅3 H_2_O (574.10)	23–100	5.27	5.97	70	85	Loss of 2H_2_O (Lattice)
	100–145	3.13	2.49	134	140	Loss of 1H_2_O (Lattice)
	145–199	3.13	3.10	186	188	Loss of 1H_2_O (Coordinated)
	199–437	32.65	33.39	419	432	Loss of 1.5Cl_2_+ C_5_H_7_N
	437–512	34.44	34.53	464	458	Loss of C_10_H_5_N_4_O leaving 2Ni residue
	512	20.45	20.52			% of Ni residue
Cd complex [Cd_2_(L)(H_2_O)(Cl_3_)] (627.48)	90–195	5.26	5.34	93 179	102	Loss of H_2_O (coordination) + 1CH_3_
	195–328	2.39	2.56	279	-	Loss of 1CH_3_
	328–800	Not assigned	88.51	643	434 482	Not assigned
Pt complex [Pt_2_(L)(H_2_O)(Cl_3_)] (792.82)	155–192	2.27	3.00	178	not assigned	Loss of H_2_O (coordination)
	192–284	1.89	2.32	224		Loss of CH_3_ group
	284–423	44.60	43.18	326 388		Loss of 1.5Cl_2_ + C_14_H_9_N_5_ leaving Pt + PtO residue
	423	49.21	49.47	-		% of Pt residue

### Electronic absorption and magnetic moment measurements

The electronic spectral data of the ligand and its metal chelates were recorded to identify the mode of electronic transitions within the desired compounds, which helps to determine the coordination geometry surrounding the metal coordination centers in the synthesized chelates. The measurements were carried out in DMF solvent in the 200–800 nm region. The absorption bands at shorter wavelengths are related to transitions within the organic ligand structure. The absorption spectrum of the **MPAQ** ligand exhibited three well-defined absorption bands appearing at 227, 330 and 340 nm, which were attributed to π→π* transitions within the ligand associated with aromatic rings, azo and azomethine moieties, respectively. The absorption band appearing at 435 nm is related to the n→π* electronic transition due to non-bonding electron pairs in C = N and/or N = N groups existing in the ligand structure^58^. Such bands afforded a significant shift in their positions in the spectra of metal complexes, confirming the involvement of the azo and azomethine groups in coordination with the metal centers.

For **MPAQ-Ni** complex, additional peaks characteristic for d-d transitions were observed, which may help to conclude the geometry around the Ni centers. The complex’s spectra showed three distinct absorption bands at 450, 540, and 726 nm, attributed to transitions which are in agreement with the Ni(II) tetrahedral transitions ³T₁(F) → ³T₁(P), ³T₁(F) → ³A₂(F), and ³T₁(F) → ³T₂(F)^59^. The calculated magnetic moment of the Ni(II) complex was calculated to be 2.87 B.M per one Ni centre, consistent with high-spin Ni^2+^ complexes.

**MPAQ-Cd** complex displaying d^10^ electronic structure with just paired electrons, there isn’t a d–d electronic transition. The spectrum exhibits ligand-based electronic transition bands, together with a charge-transfer band observed at 453 nm. The electronic spectrum did not introduce any useful evidence regarding the stereochemistry of the complex.

**Fig. 6:**
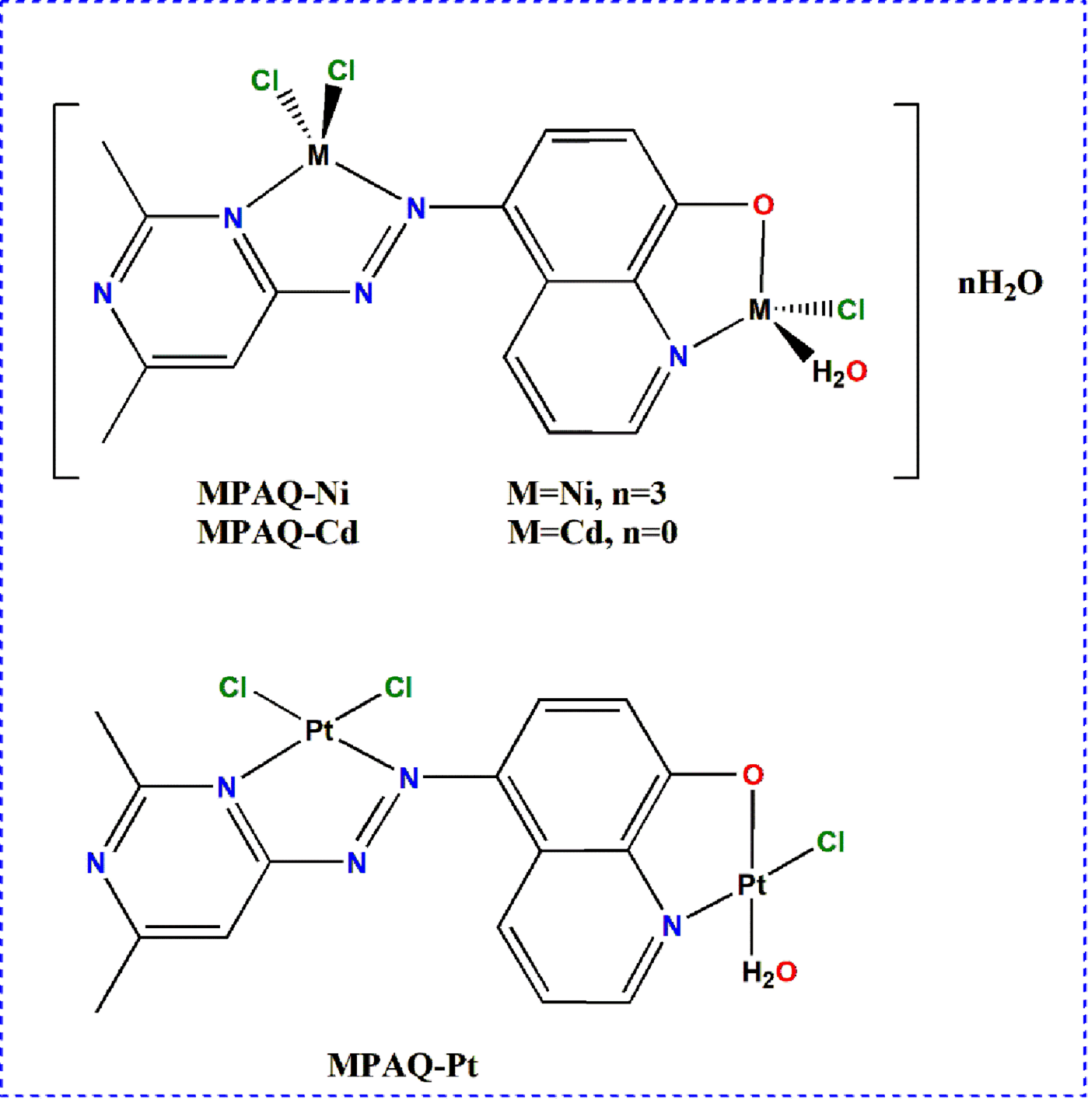
Structures proposed for the metal complexes MAPQ-Cd, MAPQ-Ni, and MAPQ-Pt.

Consistent with the transition identified by Jørgensen, the Pt(II) complex showed in its electronic spectrum a low-energy spin-allowed band at 500 nm corresponding to the transition 1A1g →A2g (v1)^60^ for [PtCl_4_]^−2^ (465 nm). The absorption band at 451 nm is attributed to a combination of ^1^A_1g_→ ^1^E_g_ (ν_3_) and MLCT transitions. These two transitions support a square planar geometry^61^. Both **MPAQ-Cd** and **MPAQ-Pt** complexes are diamagnetic, as expected. All the previous spectral and analytical results assured the structures and geometries of the current complexes as shown in Fig. 6.

### XRD pattern

**Fig. 7:**
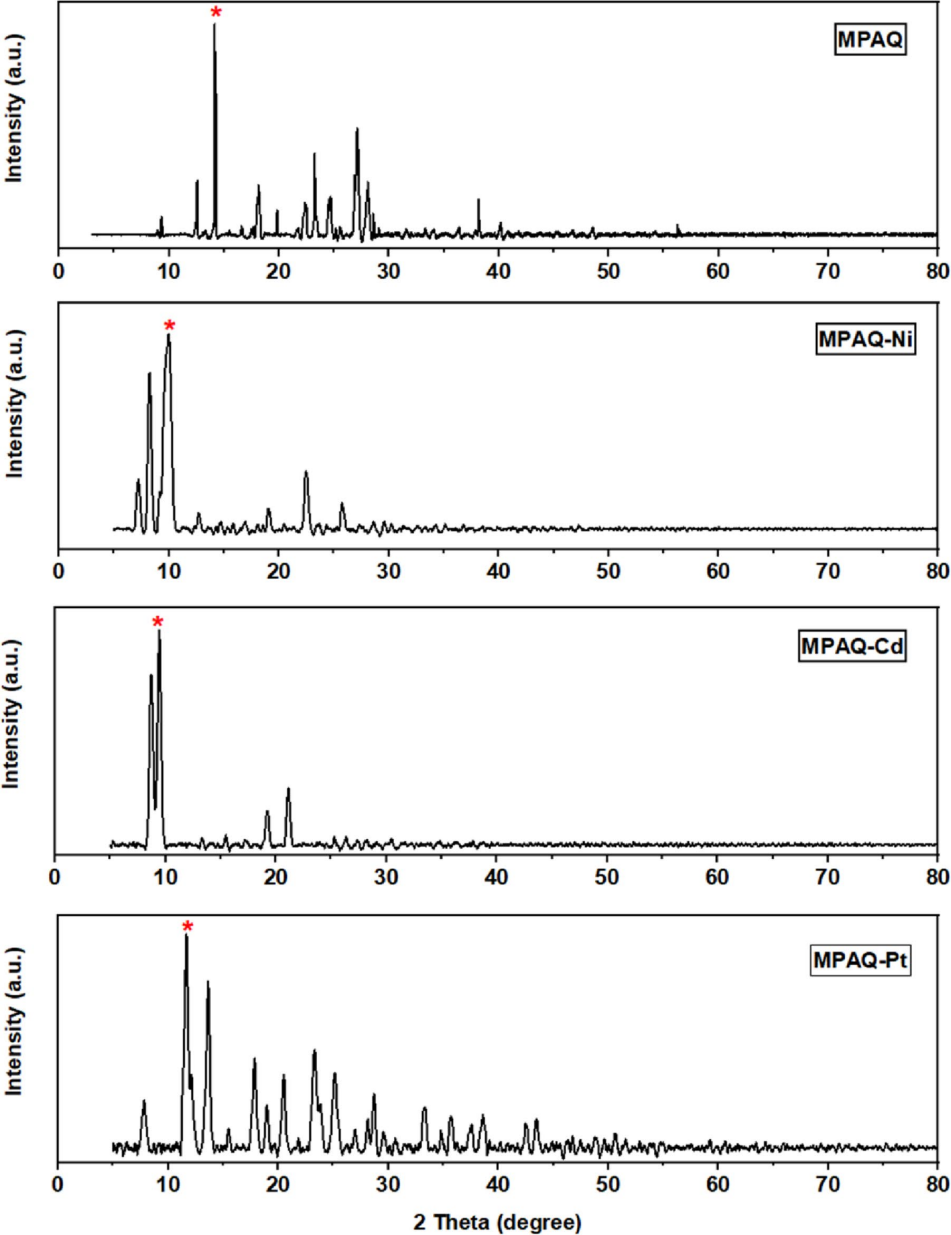
XRD pattern of **MPAQ** and its metal complexes.

Powder X-ray diffraction is a powerful characterization method for acquiring microcrystalline data on newly synthesized compounds. It is frequently employed to investigate the crystallinity and identify the crystalline system of solid-state complexes. X-ray diffraction patterns of **MPAQ** and its complexes **MPAQ-Ni**, **MPAQ-Cd** and **MPAQ-Pt** are interpreted in Fig. 7. The quantitative diffraction data, including positions, d-spacing values, and relative intensities, are summarized in Table 2. The compounds’ diffractions have been measured between 2Θ of 5 and 80^◦^. The XRD patterns were recorded using a generator with an applied voltage of 40 kV and current of 30 mA, using a Cu anode and Kα radiation (λ = 0.154060 nm). The data collected in Table 2 are gained from the peaks with higher intensity of each compound.

Precise investigation of the diffraction patterns shown in Fig. 7 shows the crystalline phase structures of all investigated compounds. It is evident that the diffraction patterns of the complexes differ from the parent ligand. This may be attributed to the emergence of new crystalline phases because of metal-ligand coordination, confirming the successful synthesis of the complexes^62^. While the **MPAQ** ligand displays sharp, intense peaks, indicative of a highly crystalline structure. After complex formation, the crystallinity decreased in the case of **MPAQ-Cd** and **MPAQ-Ni** complexes, while it largely increased in the case of **MPAQ-Pt** complex. Such behaviors suggest structural modifications resulting from the formation of the complex.

The average crystallite size (D) of the formed compounds was calculated using the Debye–Scherrer equation using the the 2θ value and FWHM of the highest intensity diffraction peak for each compound. The values were calculated and are summarized in Table 2^63^.

$$\:D=\raisebox{1ex}{$k{\uplambda\:}$}\!\left/\:\!\raisebox{-1ex}{$\beta\:cos\theta\:$}\right.$$

Microstrain (ε) and dislocation density (δ) can also be calculated by applying the following equations:

$$\:\delta\:={1/D}^{2}\:\epsilon\:=\raisebox{1ex}{$\beta\:$}\!\left/\:\!\raisebox{-1ex}{$4tan\theta\:$}\right.$$

For the peaks located at 2θ = 14.175, 9.963, 9.431, and 11.65**3**^◦^ (the sharpest peaks) for **MPAQ**, **MPAQ-Ni**, **MPAQ-Cd**, and **MPAQ-Pt**, respectively, the values of D were calculated to be 66.15, 15.01, 31.50, and 33.27 nm, representing the nanostructures of all compounds. Dislocation values were found to equal 0.228, 4.43, 1.007 and 0.9 × 10^− 3^ nm^− 2^, respectively. The lower δ values indicate the lower extent of structural irregularity, meaning high crystallinity of the compounds, which causes the size of the grains to be larger. Micro-strain values are 4.24, 26.58, 13,38 and 10.26 × 10^− 3^, respectively.

**Table 2:** XRD results of the compounds investigated.

Comp.	2θ^a^ (º)	d^b^ (Å)	Β^c^ (FWHM)	Crystallite size (nm)
MPAQ	12.532	7.05770	0.100	83.47
	**14.175**	**6.24307**	**0.121**	**69.16**
	18.149	4.88405	0.224	37.51
	22.392	3.96729	0.249	33.97
	23.251	3.82251	0.100	84.71
	24.686	3.60353	0.253	33.57
	27.123	3.28507	0.266	32.09
	28.085	3.17469	0.276	30.99
	28.645	3.11385	0.100	85.64
MPAQ-Ni	7.218	12.23724	0.303	27.44
	8.228	10.73707	0.254	32.75
	**9.963**	**8.87063**	**0.531**	**15.70**
	19.005	4.66595	0.100	84.13
	22.487	3.95069	0.384	22.03
	25.733	3.45919	0.100	85.11
MPAQ-Cd	8.704	10.15112	0.263	31.64
	**9.431**	**9.37045**	**0.253**	**32.91**
	19.228	4.61235	0.277	30.38
	21.132	4.20080	0.245	34.45
MPAQ-Pt	**11.653**	**7.58781**	**0.240**	**34.75**
	13.645	6.48418	0.226	36.98
	17.858	4.96284	0.231	36.36
	18.976	4.67305	0.234	35.95
	20.518	4.32516	0.240	35.13
	23.317	3.81184	0.100	84.72
	25.150	3.53811	0.379	22.43

^a^θ is the scattering angle ^b^d is the the spacing between crystal planes.

^c^β (FWHM) is the line width measured at half of the peak’s maximum intensity.

### Theoretical investigation

#### Geometry of the complexes

**Fig. 8:**
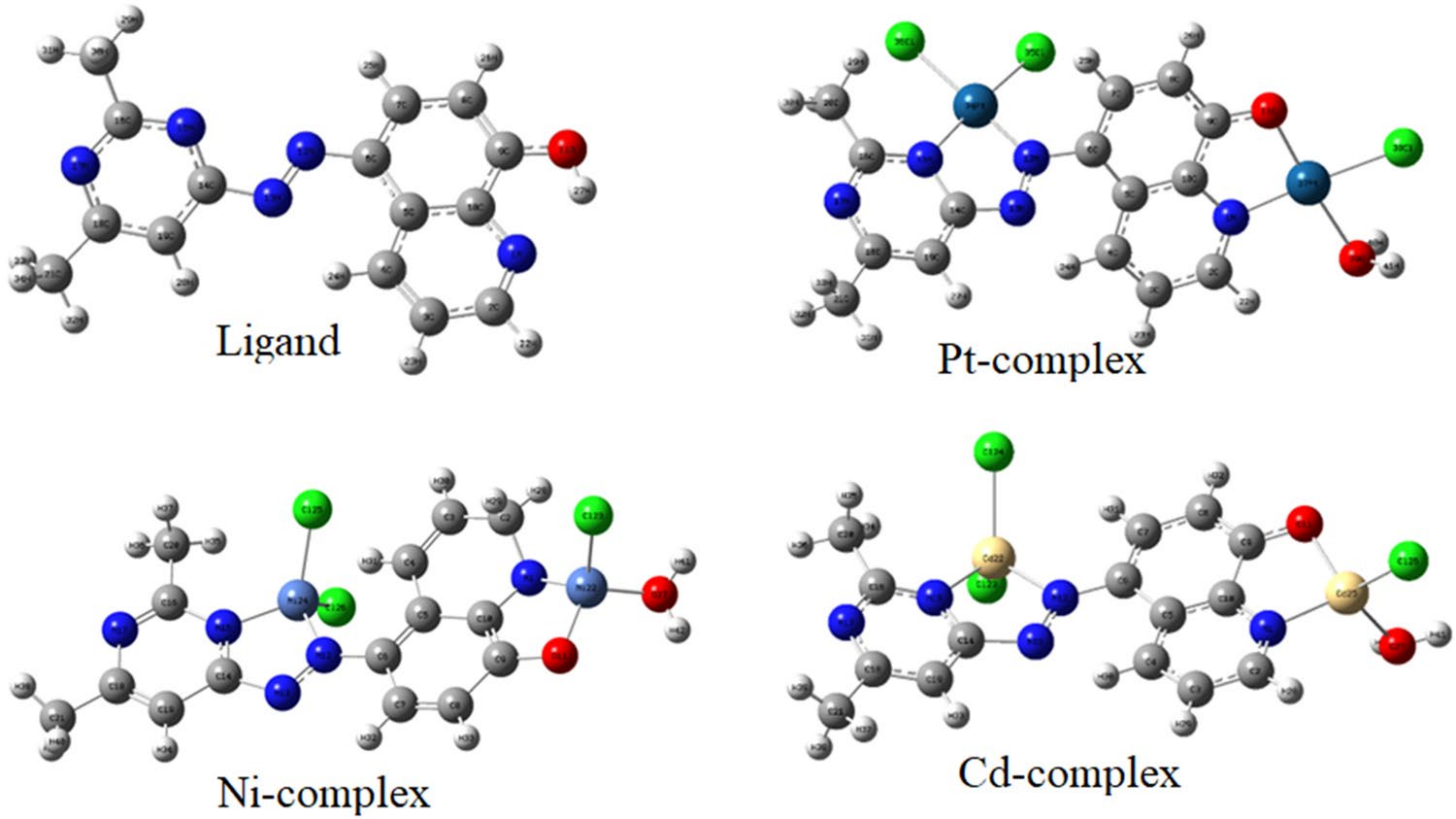
The optimized molecular geometry of the interested ligand and its metal chelates.

The B3LYP computational method, combined with 6-311 + G(d, p) for ligand atoms and LANL2DZ basis sets for metal ions were employed for the optimization of ground-state geometries of the ligand and its complexes. Tables S2 and S3 combine the bond lengths and bond angles of the ligand, in addition to the charge distribution involving the metal ions in the metal complexes. The optimized structures, including atoms number are presented in Fig. 8. In general, the bond lengths (N1-C2, N1-C10, C8-C9, C9-C10, C9-O11, N12–N13, N12-C6 and C14-N15) are longer in **MPAQ-Ni**, **MPAQ-Pt**, and **MPAQ-Cd** compounds upon comparing with the free ligand (Tables S2 and S3). The elongation in these bonds assures the ligation of the atoms N1, O11, N12, and N15 to the central metal ions. Elongation of the M–N13 and M–O20 bonds is observed in complexes during complex coordination (Fig. 8). Compared with typical M–X (X = O, N) bond lengths, the metal–ligand coordinate bonds lengths, such as M–N and M–O, are relatively longer. This suggests the minimum ionic character of such bonds. The complexes studied have metal–ligand bond lengths that are generally comparable, averaging 2.018 Å. The bond lengths of the Pt(II) and both of Ni(II) and Cd(II) ions with the donating atoms in the ligand support square planar and tetrahedral geometries with slight distortion around the central metal ions, respectively. Bond angle measurements (N1PtO11), (N1PtO39), (O11PtCl38), (Cl38PtClO39), and (N12PtCl35), (N12PtN15), (N12PtCl35), (N15PtCl36) and (Cl35PtCl36) in the **MPAQ-Pt** complex encourage that the compound adopts a distorted square planar geometry surrounding both metal ion centers. The bond angles (N1PtCl38, 177.874^°^, O11PtO39, 178.137^°^ N12PtCl35, 171.278^°^ and N12PtCl36, 173.079^°^) emphasize a clear deviation from the ideal linearity. These values confirm the distorted nature. These theoretical parameters are consistent with the experimental data, confirming the stoichiometric and structural assignments for the complexes. The bond angle values (N1NiO11), (N1NiO27), (N1NiCl23), (O11NiCl23), (O11NiO27),((Cl23NiO27), (Cl25NiCl26), (N12NiN15), (N12NiCl25), (N12NiCl26), (N15NiCl25), (N15NiCl26) and are 97.607°, 113.963°, 113.671, 114.672°, 112.918°, 104.408°, 102.242°, 90.915°, 117.214°, 115.017°, 116.693° and 115.542°, respectively, Table S2. These results agree well with a tetrahedral geometry of ligand atoms surrounding the Ni(II) center with distortion, that is matching with the experimental expectations. In the **MPAQ-Cd** complex, the calculated average bond angles surrounding the Cd(II) center is 109.293^°^ (Table S3), indicating that the compound adopts a distorted tetrahedral geometry in agreement with experimental results.

#### Mulliken population analysis

Mulliken population-based charge calculation is fundamental to the implementation of quantum chemical methods within molecular systems, as the distribution of atomic charges influences various molecular properties, including dipole moments and molecular polarizability. Additionally, the electrostatic potential surfaces have been described using it^64–66^. The calculations reveal that the net atomic charges on N1, O11, N12 and N15 atoms for the ligand is -0.412e, -0.995e, -0.615e and − 0.667e, respectively. This indicates that these atoms serve as the primary coordination sites since their electrostatic potentials are negative. Additionally, the calculations demonstrated that complexation has a significant impact on the net atomic charge distribution on the ligand’s ligating atoms. The calculated charges for the ligands show an increase in electron density at the C2, C9, and C14 atoms and a small electron deficiency at the C5, C6, and N13 atoms in all complexes. Additionally, the charge distribution at the coordination sites (N1, O11, N12, and N15) become less negative after complex formation. Charge transfer from the coordinating oxygen and nitrogen atoms of the ligand to the metal ions and the back donation from the metal ions’ d-orbital to the ligand’s π* antibonding character could account for the strong contact between the metal ions and the ligand. Complexation exerts a significant influence on the charge distribution on the ligand’s other atoms.

#### Ground-state electronic properties and global reactivity descriptors

**Fig. 9:**
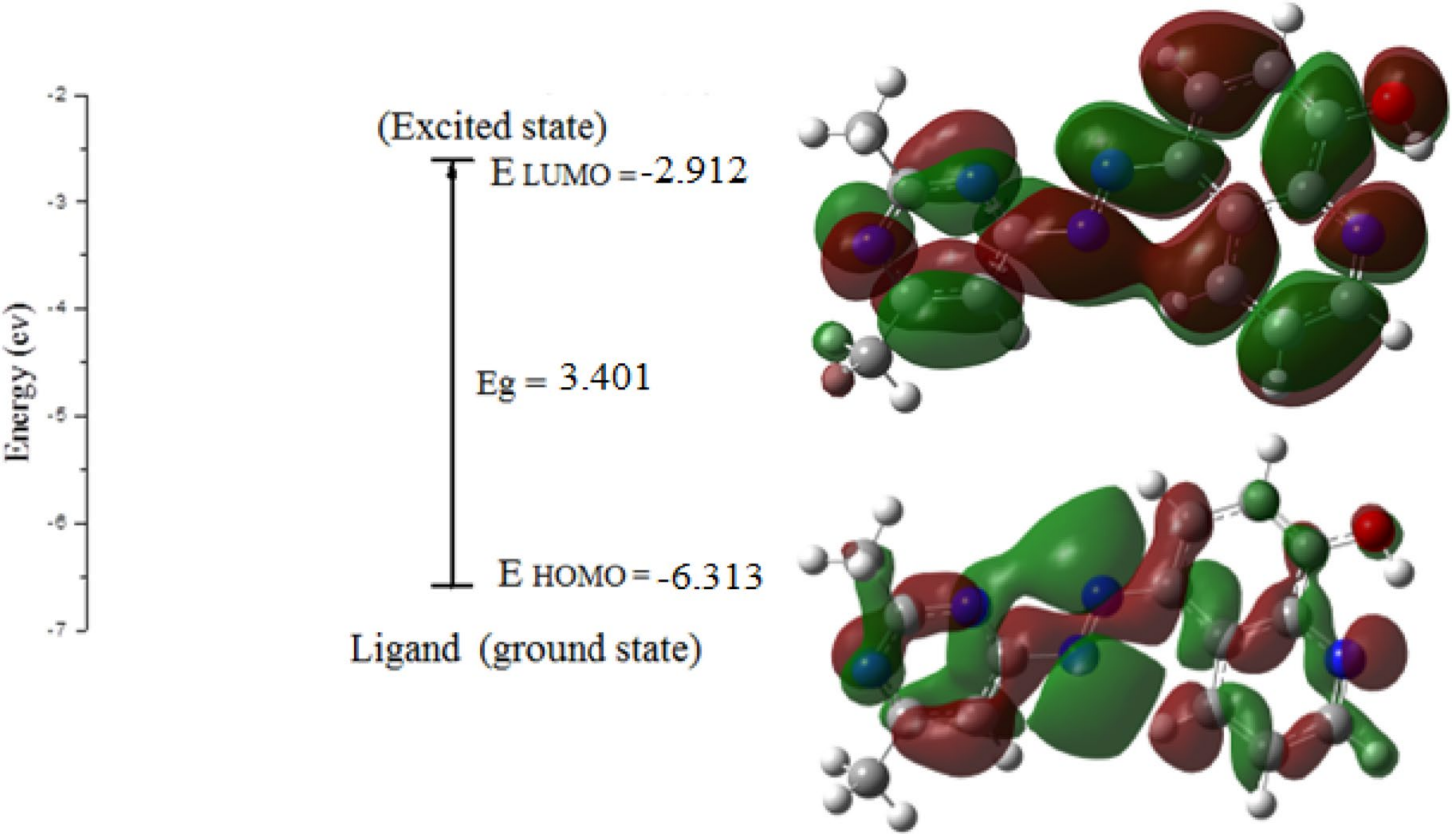
HOMO–LUMO charge density maps of the investigated ligand at the B3LYP/6-311 + G(d, p) computational level.

**Fig. 10:**
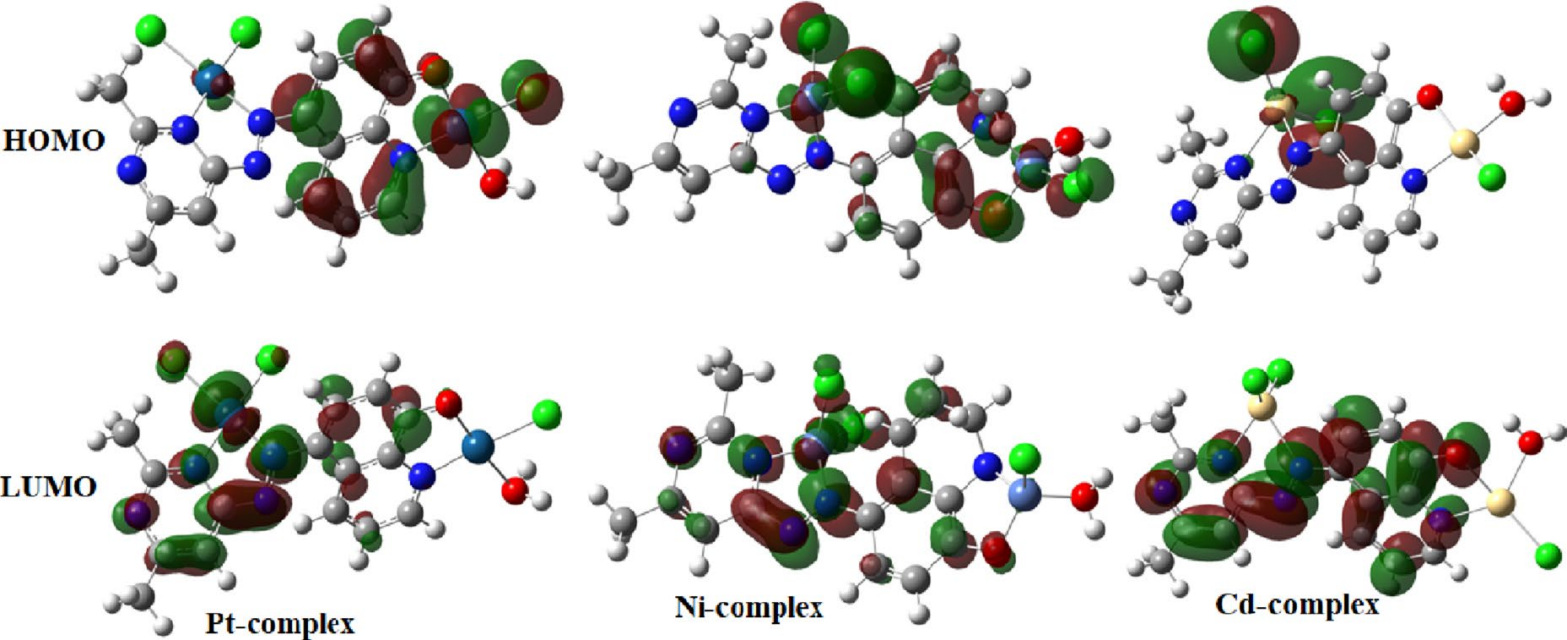
HOMO-LUMO charge density maps of the investigated complexes using the DFT/B3LYP/ LANL2DZ.

The HOMO–LUMO spatial characteristics and their corresponding energy levels, and corresponding ground state energies of the investigated ligand and its metal chelates are shown in the frontier molecular orbital (FMO) maps in Figs. 9 and 10. The analysis of the distribution and energy levels of the molecular orbitals of the molecule is generally recognized to be critical for understanding the electronic nature of molecules^67^. Frontier molecular orbitals (FMOs) are key indicators of the reactivity of compounds. The energies of the HOMO and LUMO play a crucial role in the determination of the redox behavior of the compound and in predicting the chemical stability of the synthesized complexes. Furthermore, energy gap is a significant indicator of molecular stability and hardness/softness, or Eg, between the energies of the HOMO and LUMO orbitals^68^. A soft molecule has greater polarizability compared to a hard molecule. Hardness highlights the complex’s reactivity. Consequently, a large HOMO–LUMO energy gap reflects a harder, less reactive molecule. Figure 9 shows the spatial distribution of the ligand’s HOMO and LUMO orbitals. The calculations demonstrate that the HOMO charge distribution of the examined ligand is centered on the N and O lone pairs. Additionally, the LUMO level charge distribution is significantly delocalized across the whole ligand framework with a π* antibonding character (Fig. 9). The results confirm the stability of the formed complexes is achieved by the transfer of electron density through ligand-to-metal donation from donor orbitals, accompanied by metal-to-ligand back-donation. The distribution of charge density of the donor (HOMO) and acceptor (LUMO) levels is shown in Fig. 10. The calculations indicate the presence of ligand-to-metal σ-donation accompanied by back-donation from the metal ion d orbitals to the ligand, thus stabilizing the complex formation.

#### Molecular electrostatic potential (ESP)

**Fig. 11:**
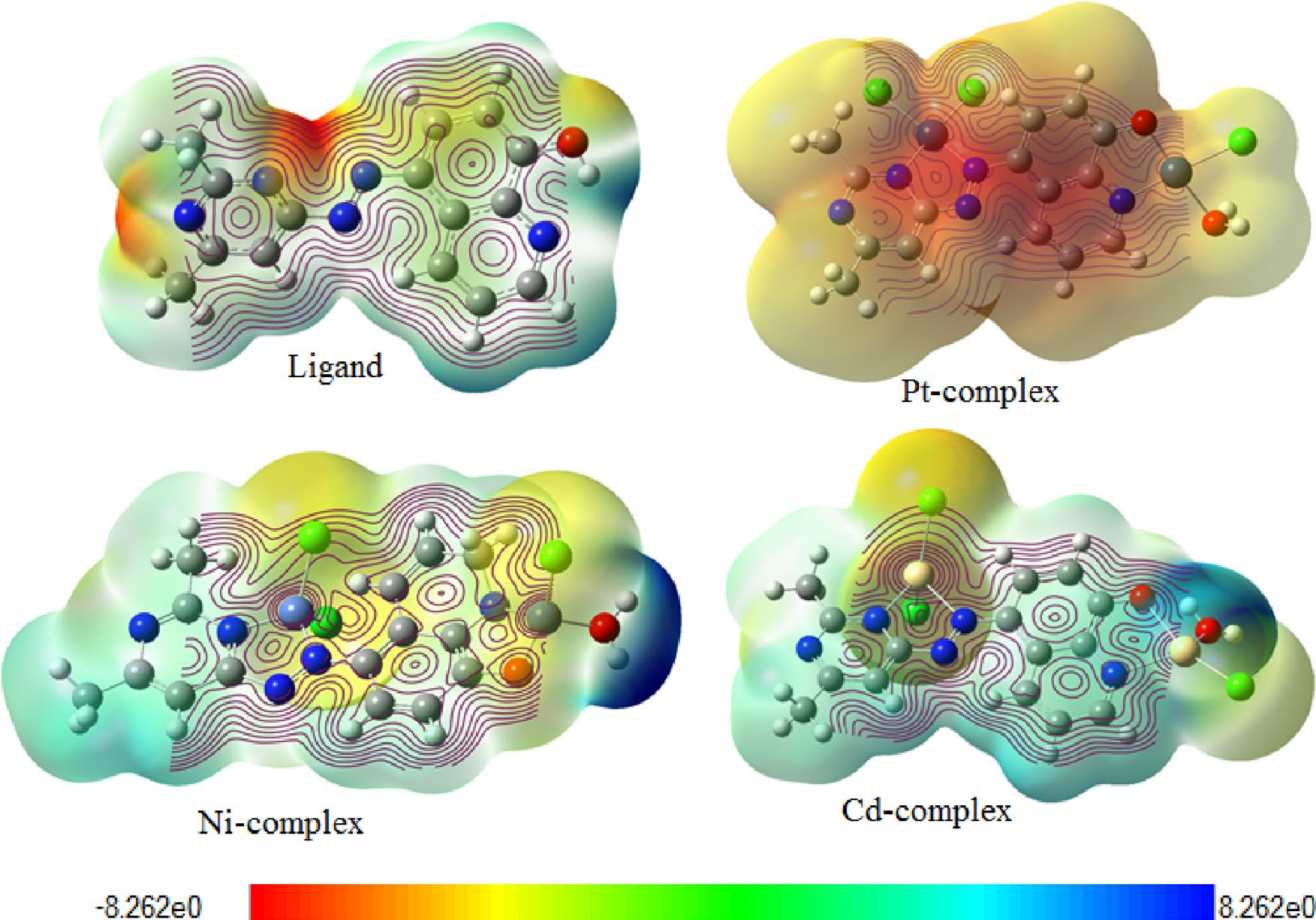
MEP graph for the investigated ligand and its metal complexes. The electron density isosurface is 0.004 a.u.

The molecular electrostatic potential (ESP) is a critical parameter for identifying the preferred sites for electrophilic or nucleophilic attack^69^. Additionally, it provides important details on non-bonded interactions^70^. The blue-colored regions on the surface correspond to positive electrostatic potential distribution. representing electrophilic reactive sites, whereas the red-colored regions indicate negative electrostatic potential distribution, highlighting the centers of nucleophilic reactivity. The investigated ligand in this work possesses a negative potential that is assigned to the nitrogen and oxygen atoms, as seen in Fig. 11. This demonstrates that the ligand possesses active coordination sites for metal ion binding and may interact with positively charged regions of the cell wall structure. However, the calculations reveal that most of the complexes under investigation have positive electrostatic potential throughout their entire skeleton, with the exception of the **MPAQ-Pt** complex, which has negative potential throughout its entire skeleton. These electronic characteristics are crucial for determining the optimal docking pose inside the binding site of the target microorganism to yield a thermodynamically stable complex. This finding aligns with the frontier molecular orbital theory of electron density distribution. From the above, it is evident that the biological behavior of the compounds can be determined by the quantum chemical characteristics confirmed by MEPs.

#### The calculated quantum chemical parameters

The mechanism of complex formation is characterized by the metal ion acting as a Lewis acid and the ligands as Lewis bases. Since metals are soft acids, the complexation process works best with a soft base ligand. The ligand exhibits EHOMO and ELUMO values of − 0.232 and − 0.107 a.u., respectively, resulting in a HOMO–LUMO energy gap (Eg) of 3.401 eV, as illustrated in Fig. 9. This observation helps explain the ligand’s stability. To evaluate the influence of solvation on the electronic properties of the ligand (**MPAQ**), additional calculations were performed IN dimethyl sulfoxide (DMSO) using the Polarizable Continuum Model (PCM). The obtained results indicate that solvent effects introduce only minor variations in the frontier molecular orbital energies and global reactivity descriptors. In particular, the HOMO energy changes from − 0.232 a.u. in the gas phase to − 0.221 a.u. in DMSO, while the LUMO energy shifts from − 0.107 a.u. to − 0.096 a.u., resulting in a slight reduction of the HOMO–LUMO energy gap (from 0.130 a.u. to 0.125 a.u.), Table 3. Similarly, the global hardness (η) and softness (σ) show only marginal variations, indicating that the intrinsic electronic stability and reactivity pattern of the molecule are largely preserved upon solvation, Table 3. Notably, the dipole moment increases significantly from 3.211 D to 4.507 D, reflecting enhanced charge separation in the polar environment. The total electronic energy also shows a minor stabilization in DMSO (− 928.639 a.u. compared to − 928.630 a.u. in the gas phase), consistent with expected solvent stabilization effects. Overall, these results demonstrate that the solvation slightly affects quantitative electronic parameters, the qualitative electronic structure, orbital distribution, and reactivity trends of MPAQ remain essentially unchanged. Therefore, the conclusions drawn from gas-phase calculations are still valid and reliable for comparative discussion.

Table 4 summarizes the ground-state frontier molecular orbital energy levels along with their partial compositions for the studied complexes in their ground-state level. The results confirm that the E_HOMO_ of the **MPAQ-Pt**, **MPAQ-Ni** and **MPAQ-Cd** complexes are − 0.238, − 0.227, and − 0.242 a.u, respectively. Compared with the E_HOMO_ value of the **MPAQ** ligand (− 0.232 a.u.), it is observed that chelation destabilizes the HOMO in all complexes except the **MPAQ–Ni** complex, the HOMO is stabilized. MPAQ–Ni shows the highest HOMO stability among the complexes, while chelation leads to LUMO stabilization in all cases. The energy gap (Eg) values confirm that the reactivity of the metal complexes is higher than that of the free ligand and the most reactive one is **MPAQ-Ni** complex. Eg has the order: **MPAQ-Ni** < **MPAQ-Pt** < **MPAQ-Cd** < **MPAQ**. The calculated high dipole moments of the complexes favor enhanced dipole–dipole interactions within biological systems. The calculations showed that **MPAQ-Pt** complex has the highest value of dipole moment. Also, the calculations show that **MPAQ-Ni** complex has the highest softness value, 25.641 a.u^− 1^. All quantum chemical descriptors confirm that **MPAQ-Ni** complex is the most reactive toward the biological target, which explains its highest bioactivity among the studied compounds. This agrees well with the experimental results.

**Table 3:** The calculated values of quantum chemical descriptors for the investigated **MPAQ** ligand in gas and solvent phases.

Parameters	MPAQ (gas)	MPAQ (Solvent)
E_HOMO_ (a.u)	-0.232	-0.221
E_LUMO_ ( a.u )	-0.107	-0.096
E_LUMO_– E_HOMO_ (a.u)	0.130	0.125
µ( a.u )	-0.170	-0.159
η ( a.u )	0.065	0.063
σ (a.u^− 1^)	15.385	15.873
ω ( a.u )	0.222	0.202
ΔNmax	2.608	2.524
Dipole (D)	3.211	4.507
TE ( a.u )	-928.630	-928.639

**Table 4:** The DFT results of binding energies and HOMO–LUMO values for the investigated ligand and its metal complexes.

Properties	MPAQ-Ni	MPAQ-Cd	MPAQ-Pt
E_HOMO_ (a.u)	-0.227	-0.242	-0.238
E_LUMO_ ( a.u )	-0.149	-0.147	-0.155
E_LUMO_– E_HOMO_ (a.u)	0.078	0.095	0.083
µ( a.u )	-0.188	-0.195	-0.197
η ( a.u )	0.039	0.048	0.042
σ (a.u^− 1^)	25.641	21.053	24.096
ω ( a.u )	0.453	0.396	0.462
Δnmax	4.821	4.052	4.679
Dipole (D)	5.514	8.743	16.633
TE ( a.u )	-1388.029	-1145.295	-1287.411

### Biological evaluation

#### Antimicrobial activity

Antimicrobial activity of the **MPAQ** ligand and selected metal complexes (**MPAQ–Ni**, **MPAQ–Cd**, and **MPAQ–Pt**) was investigated against fungi as well as Gram-positive and Gram-negative bacteria, and the findings are summarized in Table 5 . The tested compounds exhibited different degrees of activity depending on the type of the coordinated metal ion, the type of microorganism and the activity of the compounds.

The results shown in Table 5 indicate superior activity for the ligand **MPAQ** against all tested microorganisms, with inhibition zone diameters of 51, 40, 48, 52, 40, and 53 mm against *Aspergillus fumigatus*,* Candida albicans*,* Staphylococcus aureus*,* Bacillus subtilis*, and *Proteus vulgaris*, respectively.

For the 3 tested metal complexes and with respect to antifungal activity against *Aspergillus fumigatus*, the highest inhibition was observed for the **MPAQ–Cd** complex, with an inhibition zone measuring 32 mm, exceeding the reference drug ketoconazole (19 mm), while **MPAQ-Pt** showed relatively high activity (28 mm). The **MPAQ-Ni** complex was inactive against this strain. A similar pattern was observed against *Candida albicans*, where **MPAQ-Cd** exhibited strong activity (29 mm), followed by **MPAQ-Pt** (20 mm) and the least active compound is **MPAQ-Ni** (13 mm), compared with ketoconazole (21 mm).

Against Gram-positive bacteria, **MPAQ-Pt** demonstrated remarkable activity against *Staphylococcus aureus* (38 mm), surpassing gentamycin (25 mm), while **MPAQ-Cd** and **MPAQ-Ni** showed lower inhibition (23, 24 mm, respectively). Against *Bacillus subtilis*, **MPAQ-Cd** displayed strong antibacterial activity (30 mm), exceeding gentamycin (27 mm), whereas **MPAQ-Pt** and **MPAQ-Ni** showed lower inhibition (22 and 14 mm, respectively).

Regarding Gram-negative bacteria, **MPAQ-Cd** showed the highest activity against *Escherichia coli* ATCC (20 mm), followed by **MPAQ-Pt** (17 mm) and **MPAQ-Ni** (11 mm). Against *Proteus vulgaris*, moderate inhibition was observed for **MPAQ-Cd** (23 mm) and **MPAQ-Pt** (20 mm), while **MPAQ-Ni** exhibited comparatively lower activity (15 mm).

The ligand **MPAQ** is more active than its complexes against all tested strains, according to a thorough analysis of the results in a behaviour similar to closely reported compounds^71^. The decreased activity exhibited by the complexes, in contrast to the free azo compound, may result from their reduced potential to coordinate with metal ions necessary for microbial metabolic functions in addition to hydrogen bonding interactions with cellular active sites, which lead to the distribution of the normal cell cycle. The lack of activity and, thus, the limited antimicrobial effect may be further explained by the poor lipophilicity of the compounds, which hinders their diffusion across the lipid membrane^72^.

Additionally, the identity of the metal ion involved in coordination, which plays an essential role in this study, may be responsible for the various effects of the metal complexes on the microorganisms under study when comparing their activities to one another. The metal complex’s interaction with the microbe’s biomolecules, which differ according to the type of microorganism investigated, and the thickness of the cell walls, which may impede the microbe’s cell growth, also influences the differences in the complexes’ activity^73,74^.

**Table 5:** The antimicrobial efficiencies of **MPAQ** ligand and its chelates.

Compound	Inhibition zone diameter (mm)					
	Aspergillus fumigatus	Candida albicans	Staphylococcus aureus	Bacillus subtilis	Escherichia coli ATCC	Proteus vulgaris
MPAQ	51	40	48	52	40	53
MPAQ-Ni	NA	13	24	14	11	15
MPAQ-Cd	32	29	23	30	20	23
MPAQ-Pt	28	20	38	22	17	20
Ketoconazole	19	21	-	-	-	-
Gentamycin	-	-	25	27	29	26

#### Antitumor activity

**Fig. 12:**
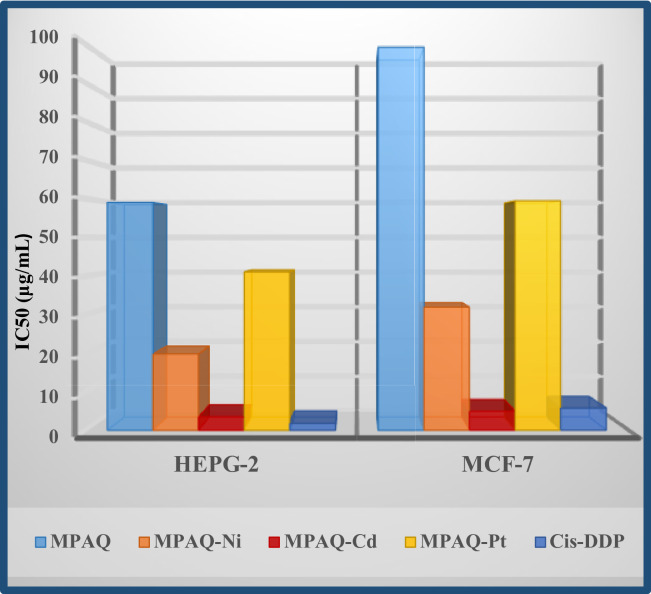
Comparative IC50 values of the tested compounds along with the standard Cis-DDP against HEPG-2 and MCF-7.

The **MPAQ** ligand and its metal complexes (**MPAQ-Ni**, **MPAQ-Cd**, and **MPAQ-Pt**) were evaluated for their in vitro cytotoxic effects against HepG-2 (liver cancer) and MCF-7 (breast cancer) cell lines by the MTT assay following 48 h of incubation. (Fig. 12; Table 6). The results were reported as IC₅₀ values (µg/mL), indicating the concentration necessary to reduce cell viability by 50% compared with the control group. The IC₅₀ values were obtained from dose–response curves correlating compound concentration with percentage cell viability. All experimental assays were repeated three times, and the data are given as mean ± standard deviation (SD).

Against the HepG-2 cell line, the **MPAQ-Cd** complex recorded the highest cytotoxic potency with an IC₅₀ value of 3.44 ± 0.12 µg/ml. The **MPAQ-Ni** complex showed moderate activity (IC₅₀ = 19.74 ± 1.06 µg/ml), whereas **MPAQ-Pt** demonstrated comparatively weaker cytotoxicity (IC₅₀ = 41.04 ± 1.08 µg/ml). The free ligand **MPAQ** displayed significantly lower activity, affording an IC₅₀ value of 59.09 ± 1.74 µg/ml.

A similar pattern was detected in the MCF-7 cell line. The **MPAQ-Cd** complex again showed the strongest cytotoxic effect (IC₅₀ = 4.91 ± 0.26 µg/ml), followed by **MPAQ-Ni** (IC₅₀ = 31.86 ± 1.59 µg/ml), while **MPAQ-Pt** exhibited lower activity (IC₅₀ = 59.54 ± 1.54 µg/ml). The parent ligand **MPAQ** demonstrated the lowest cytotoxic effect compared to the other compounds, with an IC₅₀ value of 99.34 ± 2.91 µg/ml.

Overall, complexation of **MPAQ** ligand with metal ions markedly enhanced its antiproliferative effect against both tumor cell lines studied. Among the investigated compounds, the cadmium complex demonstrated the most promising cytotoxic profile, followed by the nickel complex, whereas the platinum complex exhibited comparatively lower potency.

Based on the obtained IC₅₀ values, the order of cytotoxic activity for both HepG-2 and MCF-7 cell lines can be arranged as follows: **MPAQ-Cd** > **MPAQ-Ni** > **MPAQ-Pt** > **MPAQ**. These findings confirm the significant influence of the coordinated metal ion on cytotoxic behaviour and highlight the superior antitumor potential of the **MPAQ-Cd** complex among the studied compounds, warranting further mechanistic and biological investigations. The results of docking the tested compounds into the topoisomerase II active sites fit this order of action quite well.

**Table 6:** IC_50_ values (µg/ml) of the synthesized compounds against the HepG-2 and MCF-7.

Compound	In Vitro Cytotoxicity IC_50_ µg/ml (µM)	
	HepG-2	MCF-7
MPAQ	59.09 ± 1.74 (211.5 ± 1.74)	99.34 ± 2.91 (355.6 ± 2.91)
MPAQ-Ni	19.74 ± 1.06 (34.38 ± 1.06)	31.86 ± 1.59 (55.49 ± 1.59)
MPAQ-Cd	3.44 ± 0.12 (5.48 ± 0.12)	4.91 ± 0.26 (7.82 ± 0.26)
MPAQ-Pt	41.04 ± 1.08 (51.76 ± 1.08)	59.54 ± 1.54 (75.10 ± 1.54)
Cis-DDP	1.77 ± 0.11	5.71± 0.10

### In-silico studies

#### Molecular docking

Initially, a validation step for the docking program (MOE) was conducted to confirm the accuracy of the applied method by investigating RMSD values and how the ligands bind to the protein amino acids. Validation of this method is achieved by overlaying both the native co-crystallized ligand (blue) with GLY (green) poses. A distinct molecular redocking procedure for the co-crystallized ligand (GLY) was carried out (Fig. S2). Such a figure illustrates the superimposition of the 4fm9 co-crystallized ligand and the docked pose of the same ligand, with RMSD of 1.3253 Å. Two significant types of interactions were observed between the docked Co-crystal ligand (GLY) and both GLN 542 and LEU 592 amino acids through hydrogen bond formation, which may elucidate its antagonistic activity.

The interaction of the studied compounds (**MPAQ** ligand and metal chelates **MPAQ-Ni**,** MPAQ-Cd** and **MPAQ-Pt)** with Bacillus subtilis protein (PDB code: 3WHI) to evaluate the antimicrobial activity and also with the topoisomerase II in liver cancer (PDB code: 4FM9) that is acquired from the protein data bank (PDB) database to evaluate the antitumor activity, was studied theoretically by applying molecular docking^75,76^.

**Fig. 13:**
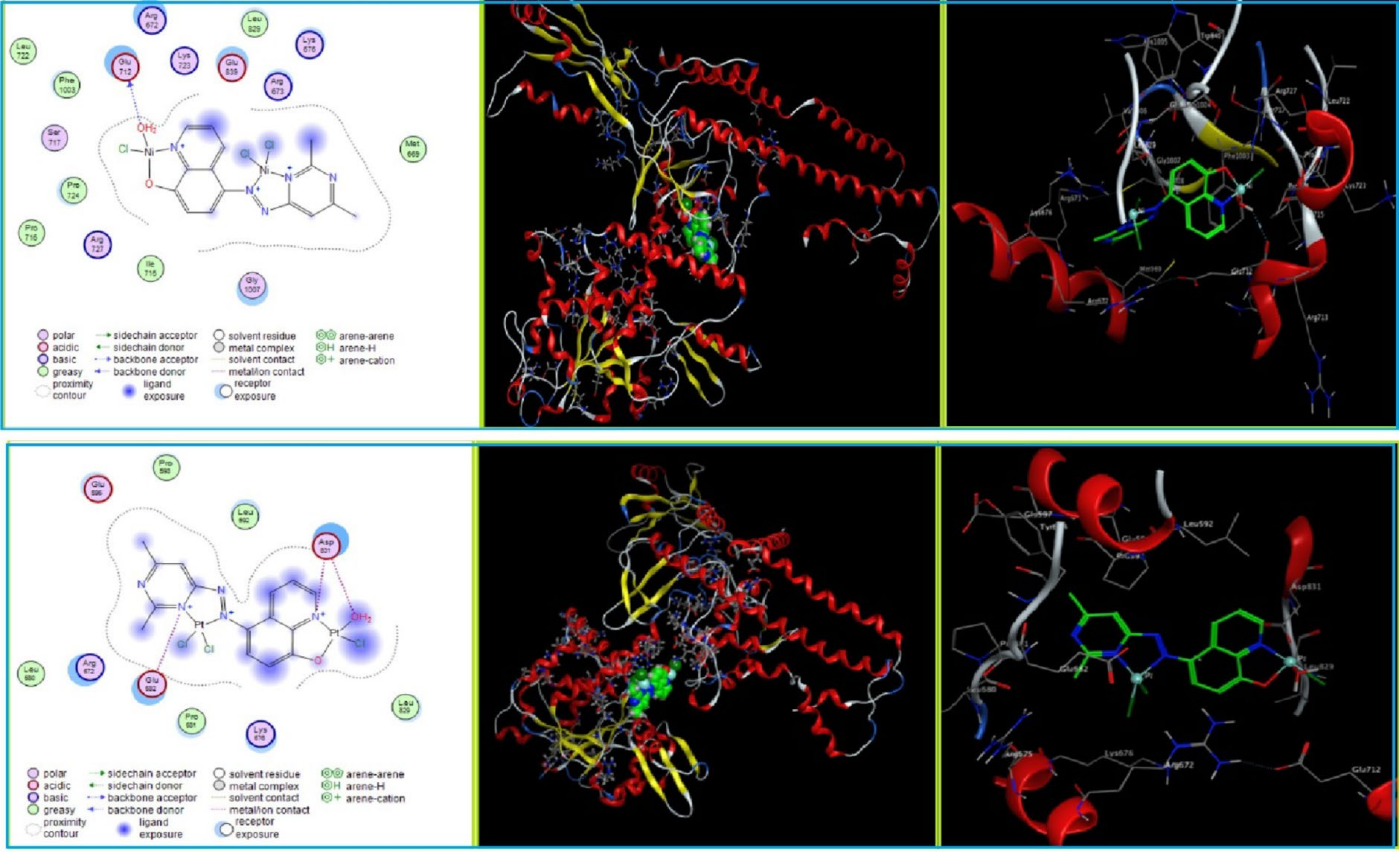
2D and 3D snaps of the binding modes of MPAQ-Ni and MPAQ-Pt into the active site of the protein with pdb code 4fm9.

**Fig. 14:**
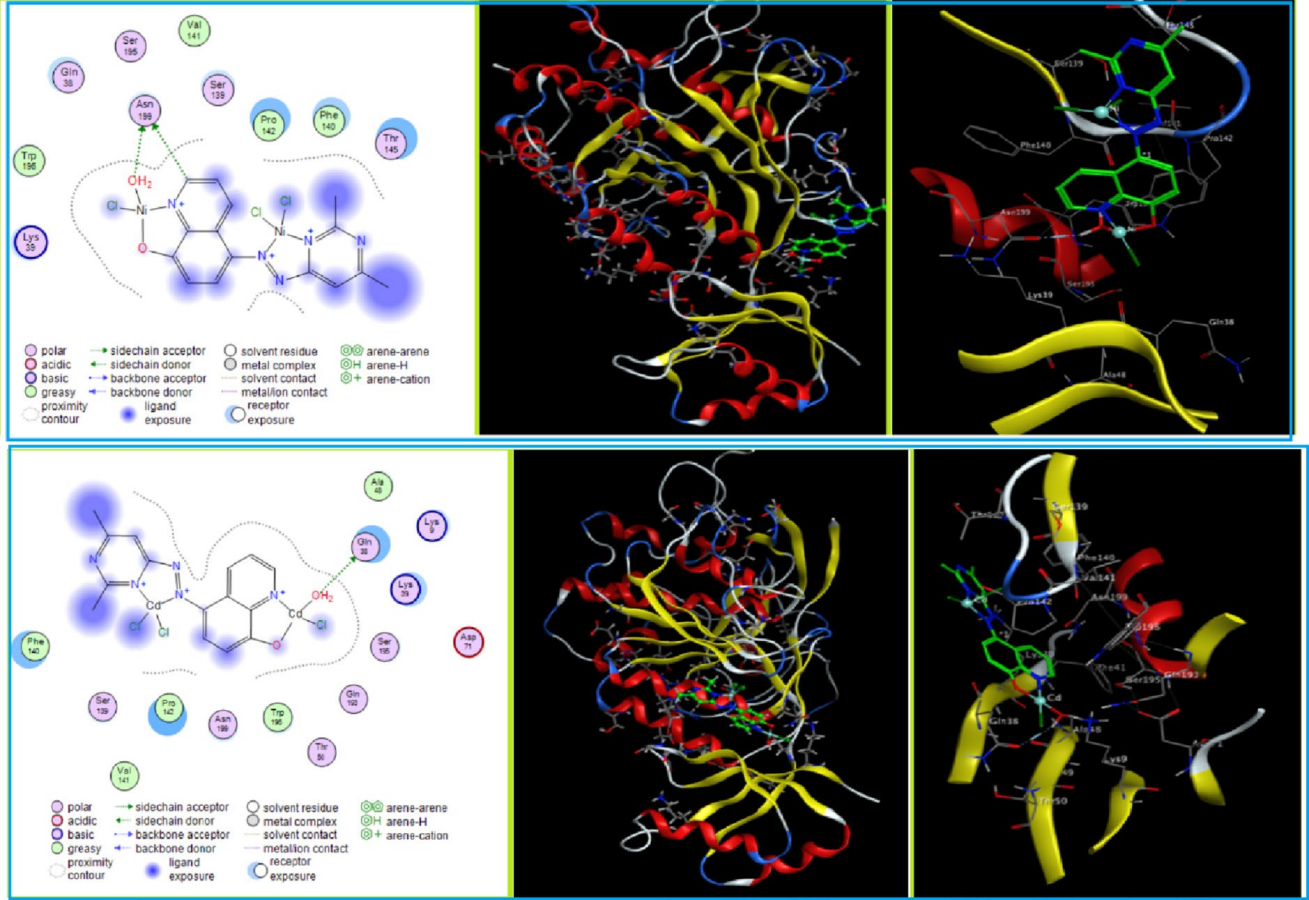
2D and 3D snaps of the binding modes of MPAQ-Ni and MPAQ-Cd into the active site of the protein with pdb code 3WHI.

Table 7; Figs. 13 and 14, S3 & S4 demonstrate the molecular interactions of the compounds of interest with 3WHI and 4FM9. For the interaction with 3WHI and from the results shown in Table 7, it is evident that the strongest binding was observed for **MPAQ** with a docking score of -5.5804 kcal/mol with pi-H interaction between the aromatic 6-membered ring and the receptor sites, GLN38 and GLN38. For the metal complexes, they yielded docking scores of -5.0172, -4.0008, and − 3.5775 kcal/mol for **MPAQ-Cd**, **MPAQ-Pt**, and **MPAQ-Ni**, respectively, in the same order from the most to the least active compound, taking into account that **MPAQ** ligand demonstrated the highest docking score value among all the studied compounds. The types of interactions of the 3 complexes with the receptor sites are H-bond type.

With 4FM9, **MPAQ-Cd** complex afforded the highest binding affinity with a docking score of -7.2015 kcal/mol followed by **MPAQ-Ni** (S **=** -6.6340 kcal/mol), **MPAQ-Pt** (S **=** -6.4641 kcal/mol) and lastly **MPAQ** (S **=** -6.0632 kcal/mol). **MPAQ-Cd** interacted with the protein sites by ionic and H-bond types, while the interaction in **MPAQ-Ni** is hydrogen bond type only, and in **MPAQ**-**Pt** is ionic bond.

Notably, the bond lengths for the interactions between the amino acid residues and the docked compounds are generally, in most cases, less than or equal 3.5 Å, indicating an appropriate docking pose^77^.

Docking study of Amsacrine, as a reference drug, which is known as a Topoisomerase II inhibitor, with 4fm9 also showed a docking score of -7.1095 kcal/mol, Fig. S5 (reported value is -6.855 kcal/mol^78^, which is close to that of **MPAQ-Cd**, supporting its promising activity as an anticancer agent.

**Table 7:** Molecular docking results of the ligand MPAQ and its complexes with Bacillus subtilis protein (PDB code: 3WHI) and topoisomerase II in liver cancer (PDB code: 4FM9).

Compound		Ligand moiety	Receptor site	Type of interaction	Distance (A^o^)	E (kcal/mol)	Docking score (kcal/mol)
MPAQ	3WHI	6-ring	CG GLN 38 (A)	pi-H	4.10	-0.6	-5.5804
		6-ring	CD1 GLN 38 (A)	pi-H	4.52	-0.9	
	4FM9	O16	O ASP 683 (A)	H-donor	2.84	-2.2	-6.0632
		N18	N LEU 592 (A)	H-acceptor	3.23	-2.3	
		6-ring	CG GLN 544 (A)	pi-H	4.14	-0.6	
MPAQ-Ni	3WHI	C2	OD1 ASN 199 (A)	H-donor	3.46	-0.6	-3.5775
		O39	OD1 ASN 199 (A)	H-donor	2.86	-16.3	
	4FM9	O39	O GLU 712 (A)	H-donor	3.14	-1.6	-6.6340
MPAQ-Cd	3WHI	O39	OE1 GLN 38 (A)	H-donor	3.21	-6.3	-5.0172
	4FM9	O 39	OD1 ASP 831 (A)	H-donor	2.68	-14.0	-7.2015
		O39	OD2 ASP 831 (A)	H-donor	3.20	-1.3	
		N1	OD1 ASP 831 (A)	ionic	3.41	-2.3	
		N1	OD2 ASP 831 (A)	ionic	3.34	-2.6	
		N17	OE1 GLU 712 (A)	ionic	2.99	-4.5	
		N20	OE1 GLU 712 (A)	ionic	2.88	-5.3	
		O39	OD1 ASP 831 (A)	ionic	2.68	-7.0	
		O39	OD2 ASP 831 (A)	ionic	3.20	-3.2	
MPAQ-Pt	3WHI	O38	O SER 139 (A)	H-donor	2.91	-12.3	-4.0008
	4FM9	N1	OD1 ASP 831 (A)	ionic	3.49	-1.9	-6.4641
		N1	OD2 ASP 831 (A)	ionic	2.74	-6.5	
		N20	OE2 GLU 682 (A)	ionic	3.77	-1.0	
		O38	OD2 ASP 831 (A)	ionic	3.79	-1.0	
Co-crystal ligand(GLY)	4FM9	O2	O GLN 542 (A)	H-donor	3.21	-0.7	-3.9113
		O1	N LEU 592 (A)	H-acceptor	3.28	-1.3	
Amsacrine	4FM9	N8	O LYS 701 (A)	H-donor	3.08	-3.9	-7.1095
		O6	NH1 ARG 675 (A)	H-acceptor	2.91	-5.2	
		6-ring	CA SER 591 (A)	pi-H	4.22	-0.6	
		6-ring	N LEU 592 (A)	pi-H	4.37	-0.8	
		6-ring	CA TYR 684 (A)	pi-H	3.88	-0.6	

#### In silico ADME predictions

In addition to testing the efficacy of strong chemicals and their capability to reach the target in a biologically active form, modern drug development requires conducting various clinical trials with humans, animals, and cells. Unfortunately, such tests are often quite expensive and pose a danger to participants’ health. A viable approach involves the computational analysis of absorption, distribution, metabolism, and excretion (ADME) profiles of drug candidates^79^. At the same time, computer modeling helps to supplement conventional tests by providing rapid and accurate predictions. In addition, computer models allow optimizing lead compounds to be turned into drugs for patients because they predict the properties related to pharmacokinetics, physicochemical properties, and the medicinal chemistry principles of small-molecule compounds.

SwissADME allows evaluating the ADME properties of small compounds and drug candidates and provides relevant information that enables assessing risks from the very beginning of the process of drug development. Drug-likeness means a set of molecular characteristics and attributes that allow one to find out whether an unknown molecule can be considered similar to known drugs. Such properties include hydrophobicity, electronic structure, hydrogen bond donors/acceptors, and the molecule’s size and flexibility, among others. SwissADME provides a “BOILEDegg evaluation” to predict the gastrointestinal absorption (HIA) and P-glycoprotein (P-gp) efflux/retention of the molecules. The term “BOILEDegg” is associated with a specific technique for assessing HIA depending on the molecule’s location in the WLOGP versus TPSA plot^80^. Moreover, it is possible to predict BBB passage and whether a particular compound works as a substrate or CYPs inhibitor. It should be noted that false positives are often detected when testing small molecules using biochemical tests can be accurately predicted^81^.

**Fig. 15:**
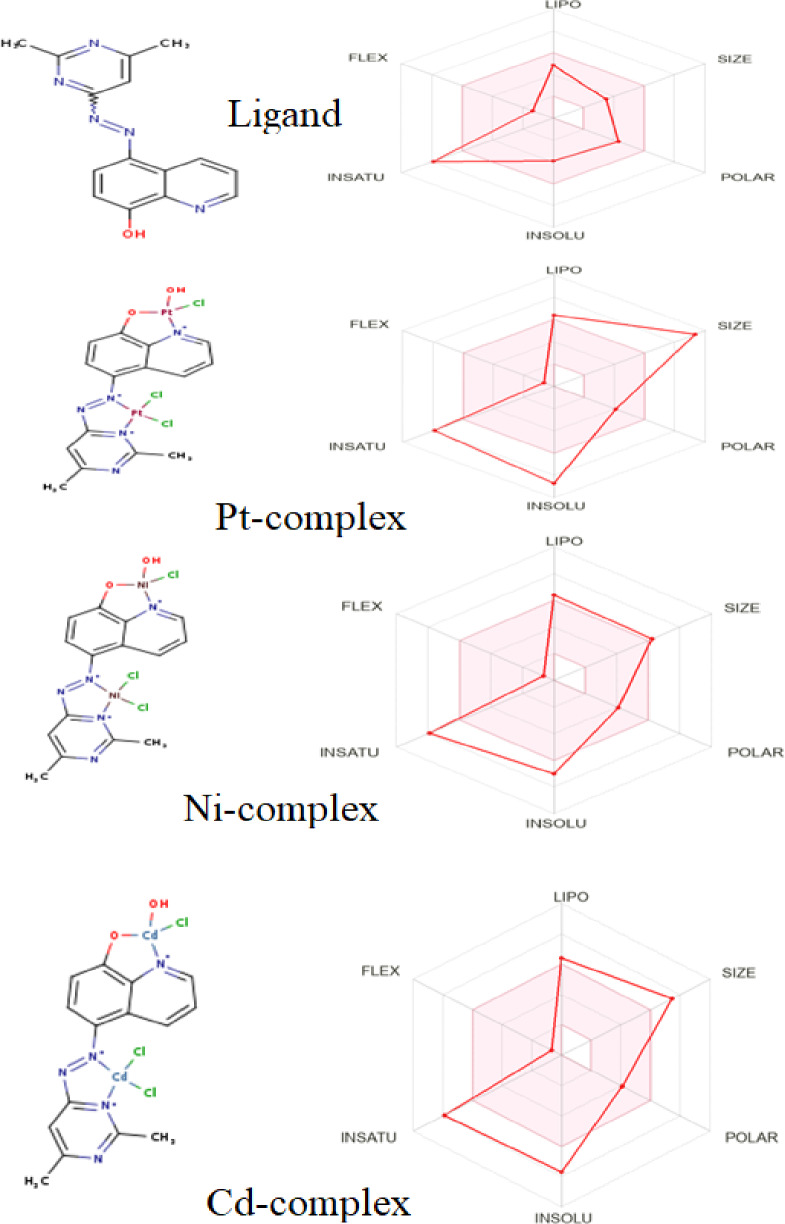
The bioavailability radar diagrams of the designed compounds, evaluated via the SwissADME web tool.

Figure 15 illustrates the bioavailability radar, which visually depicts the drug-likeness characteristics of an orally active substance. Each point on the hexagonal drug-likeness chart corresponds to a specific factor that indicates a drug’s bioavailability profile. The ideal range for every characteristic (lipophilicity: XLOGP3 falls between − 0.7 and + 5.0; size: molecular weight ranges between 150 and 500 g/mol; polarity: TPSA stays within 20 to 130 Å; solubility: log S remains below 6; saturation: the proportion of carbons in sp3 hybridization is at least 0.25; and flexibility: there are at most 9 rotatable bonds), Table 8. The red, distorted hexagon shaded in pink depicts the drug-likeness features, which for all substances comply with the criteria of a bioavailable drug.

**Table 8:** Physicochemical characteristics of the compounds (MW = molecular weight; MR = molecular refractivity; TPSA = topological polar surface area).

Molecule	Formula	M.W g/mol	#Heavy atom	#Aromatic heavy atoms	Fraction Csp3	#Rotatable bonds	#H-bond Acceptors	#H-bond donors	MR	TPSA Ǻ
MPAQ	C_15_H_13_N_5_O	279.30	21	16	0.13	2	6	1	79.91	83.62
MPAQ-Ni	C_15_H_13_Cl_3_N_5_Ni_2_O_2_	519.04	27	16	0.13	1	4	1	104.66	76.93
MPAQ-Cd	C_15_H_13_Cd_2_Cl_3_N_5_O_2_	626.48	27	16	0.13	1	4	1	104.66	76.93
MPAQ-Pt	C_15_H_13_Cl_3_N_5_O_2_Pt_2_	791.81	27	16	0.13	1	4	1	104.66	76.93

#### Drug-likeness

The chemicals that show pharmacological or therapeutic effects should possess several favourable characteristics, such as pharmacokinetic ADMET features and drug-likeness attributes. Besides that, there are filters included in the software for prediction of ADMET properties, such as Lipinski’s five rules^82^, Ghose filter^83^, Egan filter^84^, Veber filter^85^, and Muegee filter^86^. Table 9 describes the anticipated ADME properties of compounds designed in this study. Table 9 reveals that the predicted molecules exhibit minimal violations of the specified criteria and align well with the rules. Therefore, the designed compounds will probably exhibit drug activity.

**Table 9:** SwissADME-based drug-likeness evaluation of the studied molecules.

Molecule	Lipinski Violations	Ghose violations	Veber violations	Egan violations	Muegge violations
MPAQ	Yes: 0 violation	Yes	Yes	Yes	Yes
MPAQ-Ni	Yes: 1 violation: MW > 500	No:1 violation: MW > 480	Yes	Yes	No:2 violation: MW > 600, XLOGP3 > 5
MPAQ-Cd	Yes: 1 violation: MW > 500	No:1 violation: MW > 480	Yes	Yes	No:2 violation: MW > 600, XLOGP3 > 5
MPAQ-Pt	Yes: 1 violation: MW > 500	No:1 violation: MW > 480	Yes	Yes	No:2 violation: MW > 600, XLOGP3 > 5

#### Medicinal chemistry evaluation of the molecules, including (PAINS, Brenk alerts, lead-likeness, and synthetic accessibility analyses)

Pan-assay Interaction Interference Compounds (PAINS) identify the presence of false-positive biological activities in assays. This is where the biological activity of a compound is considered as being misleading in case of a positive PAINS warning. Brenk’s selection model^87^ expanded the scope of lead optimization opportunities through a novel selection model by focusing on molecules with lower molecular weight and less hydrophobic compared to compounds that satisfy “Lipinski’s rule of 5”. Findings of PAINS alerts are presented in Table 10. A biological activity assay can be used to identify compounds having positive biological activities, but none of the compounds gave positive Brenk and PAINS warnings^88^.

All designed compounds presented in Table 10 showed a bioavailability score of 0.55, indicating the degree of activity of the designed compounds. According to some researchers, a molecule should be classified as active in case the score is greater than 0, moderate in case the score ranges from − 5.0 to 0.00, while inactive for scores below − 5.0^89,90^. All the designed compounds proved to be synthetically accessible and highly bioactive^91^.

**Table 10:** Medical chemistry evaluation of the molecules.

Molecule	PAINS alerts	Brenk alerts	Lead likeness Alerts	Synthetic Accessibility	Bioavailability Score
MPAQ	0	0	Yes	2.69	0.55
MPAQ-Ni	0	0	No21 violations: MW > 350 XLOGP3 > 3.5	4.33	0.55
MPAQ-Cd	0	0	No:2 violations: MW > 350 XLOGP3 > 3.5	4.39	0.55
MPAQ-Pt	0	0	No:2 violations: MW > 350, XLOGP3 > 3.5	4.47	0.55

#### P-glycoprotein and CYP enzyme activity prediction

The SwissADME tool provides the capability to predict if a compound behaves as a substrate or inhibitor of cytochrome P450 (CYP) isoenzymes involved in drug metabolism, as well as whether it serves as a substrate for P-glycoprotein (P-gp)^92^. The models determine if the compound in question will inhibit or serve as a substrate for a specific CYP. The effects of the newly developed compounds on enzymes involved in drug metabolism, such as CYP1A2, CYP2C9, CYP2C19, CYP3A4, and CYP2D6, were considered, as shown in Table 11. It was found that all designed molecules inhibit CYP1A2. According to SwissADME’s predictions, all molecules do not inhibit CYP2C9, while all metal complexes inhibit CYP2C19 and CYP2D6. In contrast, candidates (ligands, Pt and Cd-complexes) do not inhibit CYP3A4, as indicated in Table 11. Additionally, it was demonstrated that all metal complexes are substrates for P-gp.

#### Human Intestinal Absorption (HIA) and Blood–Brain Barrier (BBB) Permeation

**Fig. 16:**
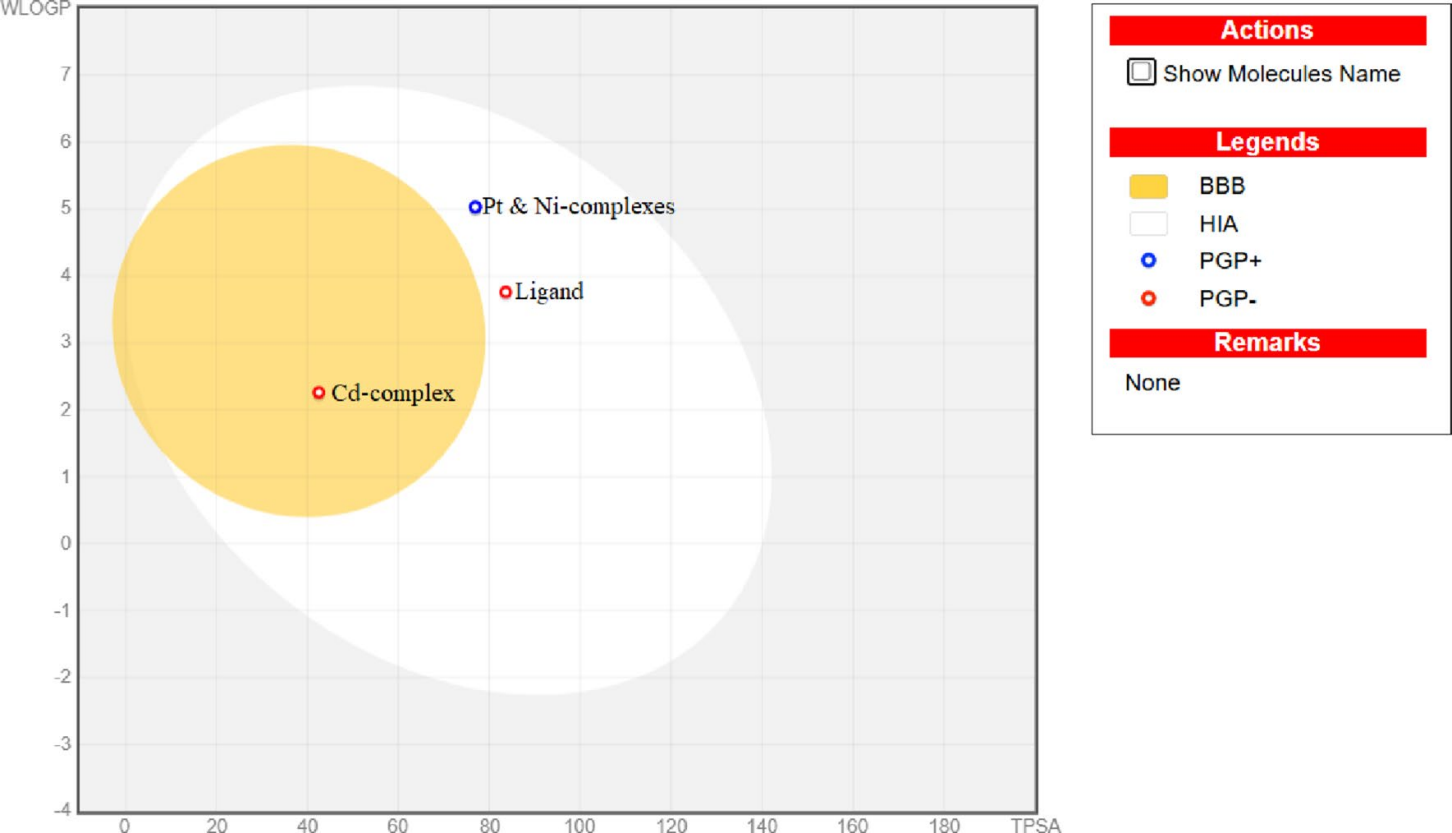
The BOILED-Egg diagram enables the prediction of passive human intestinal absorption (HIA), central nervous system permeability across the BBB, and P-gp–mediated transport properties of the examined compounds.

Human intestinal absorption and blood–brain barrier permeation are related to the functional kinetics of P-gp and CYP enzymes. The “BOILEDegg” application (Fig. 16) from SwissADME allows for the assessment of HIA based on how the molecules are arranged in the WLOGP–TPSA coordinate space. The yellow (yolk) area of the “BOILEDegg” signifies a high likelihood of brain penetration, while the white area indicates a high likelihood of passive absorption through the gastrointestinal tract. There is no disagreement between the white areas and the yolk. Additionally, data points are shown in red when they are not substrates of P-gp (PGP−), whereas they are shown in blue when they are actively transported out by P-gp (PGP+).

**Table 11:** HIA and BBB depending on the water solubility and lipophilicity of the drug.

Molecule	GI absorption	BBB permeant	Pgp substrate	CYP1A2 inhibitor	CYP2C19 inhibitor	CYP2C9 inhibitor inhibitor.	CYP2D6 inhibitor	CYP3A4 inhibitor	Logkp Skin permeation Cm/s
MPAQ	High	No	No	Yes	No	No	No	No	-5.85
MPAQ-Ni	High	No	Yes	Yes	Yes	No	Yes	Yes	-5.42
MPAQ-Cd	High	No	Yes	Yes	Yes	No	Yes	No	-6.07
MPAQ-Pt	High	No	Yes	Yes	Yes	No	Yes	No	-7.08

SwissADME incorporates two topology-based methods to estimate aqueous solubility. The first employs the ESOL model^93^, while the second is adapted from the work of Ali et al.^94^. Additionally, a third solubility predictor was developed by SILICOS-IT. All predicted values utilize the decimal logarithm of molar solubility in water (log S). The ligand molecule is determined to be soluble, whereas the metal complexes exhibit poor solubility according to the ESOL and Ali et al. models. In contrast, SILICOS-IT predicts that the ligand and Ni-complex are moderately soluble, while the Pt and Cd-complexes remain poorly soluble, as shown in Table 12. Lipophilicity, evaluated through consensus log P, indicates that ligand moieties are the most lipophilic (consensus log *P* = 2.98), whereas Ni complexes are the least lipophilic (consensus log *P* = 1.81). The consensus log P represents the average of all log P values assessed using various lipophilicity criteria, as detailed in Table 13.

**Table 12:** Water solubility assessment of the interested compounds.

Compound	ESOL				Ali				SILICOS- IT			
	Log S (ESOL)	Solubility		Class	Log S (Ali)	Solubility		Class	Log S (SILICOS)	Solubility		Class
mg/ml		mol/L	mg/ml			mol/L	mg/ml			mol/L	mol/L	
MPAQ	-3.91	3.42e-02	1.22e-04	Soluble	-4.45	9.88e-03	3.54e-05	Moderately soluble	-5.77	4.78e-04	1.71e-06	Moderately soluble
MPAQ-Ni	-7.02	4.94e-05	9.52e-08	Poorly soluble	-7.08	4.30e-05	8.29e-08	Poorly soluble	-5.92	6.21e-04	1.20e-06	Moderately soluble
MPAQ-Cd	-7.69	1.29e-05	2.05e-08	Poorly soluble	-7.08	5.19e-05	8.29e-08	Poorly soluble	-6.16	4.38e-04	7.00e-07	Poorly soluble
MPAQ-Pt	-8.71	1.53e-06	1.946e-09	Poorly soluble	-7.08	6.56e-05	8.29e-08	Poorly soluble	-6.48	2.63e-04	3.33e-07	Poorly soluble

**Table 13:** Lipophilicity assessment of the interested compounds.

Comp.	iLogP	xLogP3	wLogP	MLogP	Silicos-ITLogP	`Consensus LogP
MPAQ	2.84	3.03	3.76	1.74	3.55	2.98
MPAQ-Ni	0	5.70	5.03	1.26	-2.94	1.81
MPAQ-Cd	0	5.70	5.03	1.26	-2.28	1.94
MPAQ-Pt	0	5.70	5.03	1.26	-1.26	2.14

## Conclusion

We succeeded in the current work in the synthesis and structure confirmation of one azo ligand from 4-amino-2,6-dimethylpyrimidine and 8-hydroxyquinoline. Ni(II), Cd(II) and Pt(II) chelates incorporating this ligand were designed and obtained. Spectral and analytical tools assured successful formation of the target compounds with isolation of the 3 metal chelates in bimetallic form. Among the spectral tools used were the IR spectra that assured evidence for the chelation of the ligand to the metal ions through a monobasic tetradentate binding mode. Elemental analysis, mass spectra and TGA led to the conclusion of the complexes’ formula as [Ni_2_(MPAQ)(H_2_O)Cl_3_]⋅3H_2_O, [Cd_2_(MPAQ)(H_2_O)Cl_3_] and [Pt_2_(MPAQ)(H_2_O)Cl_3_]. Magnetic moment and UV-Vis spectral analysis assured the formed nickel chelate to have a tetrahedral arrangement. Cd(II) complex was found to be tetrahedral, and Pt(II) complex is square planar. Antimicrobial studies demonstrated significant antibacterial activities of all the tested compounds, with an exceptional effect of the ligand compared to the measured standard drugs. Anticancer efficacy of tested compounds against HepG-2 and MCF-7 showed variable activities of the tested compounds, with the highest activity shown by Cd(II) complex (IC_50_ of 3.44 ± 0.12 µg/ml toward HepG-2 and 4.91 ± 0.26 µg/ml against MCF-7 cell lines). Density functional theory (DFT/B3LYP/6-311 + G (d, p)/LANL2DZ) calculation demonstrated that the metal complexes possess a narrow energy gap, indicating enhanced chemical reactivity and a higher propensity to interact with electron-accepting biological targets. Furthermore, the calculated dipole moments of metal complexes suggest optimized dipole–dipole interactions, which are essential for stable complexation within microbial systems. These computational results can be employed for new potent therapeutic candidates, effectively bridging the gap between empirical observation and targeted molecular design.

## Supplementary Information

Below is the link to the electronic supplementary material.

Supplementary Material 1

## Data Availability

All data generated or analysed during this study are included in this published article [and its supplementary information files.
