# Supplementary material for: Structural investigation, theoretical and biological studies of 8-hydroxyquinoline azo ligand and its metal chelates
Source: Sci Rep. 2026 Jun 22;16:19370. doi: 10.1038/s41598-026-58448-4 (PMC13287710; doi:10.1038/s41598-026-58448-4)
Supplement: Supplementary file 1 — Supplementary Material 1 [file 41598_2026_58448_MOESM1_ESM.docx]

**Structural Investigation, Theoretical and Biological Studies of 8-Hydroxyquinoline Azo Ligand and its Metal Chelates**

**Mohamed M. Elkhouly*, Faten M. Atlam, Eman A. Bakr, Mohamed Gaber*****, Hoda A. El-Ghamry***

^1^Chemistry Department, Faculty of Science, Tanta University, Tanta 31527, Egypt

* Corresponding authors e-mail addresses: [mohammed.arafa@science.tanta.edu.eg](mailto:mohammed.arafa@science.tanta.edu.eg) (M.M. Elkhouly), [mabuelazm@science.tanta.edu.eg](mailto:mabuelazm@science.tanta.edu.eg) (M. Gaber), [hoda.elghamri@science.tanta.edu.eg](mailto:hoda.elghamri@science.tanta.edu.eg) (H.A. El-Ghamry)

**Section S1. Instruments and methods**

Melting points were measured on a Gallenkamp melting point apparatus without corrections. The infrared spectra were recorded on a Perkin-Elmer FTIR 1430 spectrophotometer using the KBr disk technique. Magnetic susceptibility of the paramagnetic solid chelates was recorded at ordinary temperature using a magnetic susceptibility instrument applying Gouy's technique using Hg[Co(SCN)_4_] as a calibrant. Thermogravimetric analysis (TGA) of the novel solid complexes was measured using TG-50-Schimadzu thermogravimetric analyzer under 10 ^°^C/min heating rate and N_2_ atmosphere within a temperature ranging from 25-1000 ^°^C. The ^1^H-NMR spectra were captured at 25 °C in DMSO-d^6^, with TMS serving as an internal standard using a Bruker AC spectrometer operating at 400 MHz, and chemical shifts are reported in ppm as δ values. Mass spectra were measured on a Finnigan MAT 8222 EX mass spectrometer at 70 eV. The molar conductance at room temperature is determined using the Hanna 8733 conductivity meter. A Perkin-Elmer 1430 IR spectrometer records the infrared spectra between 4000 and 400 cm^-1^ using the KBr disc technique. Element analysis (C, H, N) was also carried out at the Regional Center for Mycology and Biotechnology (RCMB), Al-Azhar University, and the values were found to be within ± 0.4% of the theoretical ones unless otherwise indicated. PXRD diffraction of the separated compounds was investigated using RIGAKU Ultima IV XRD with Cu kα radiation (λ=1.5418 A^°^) with (2Ɵ^°^) range from 5 to 70^°^ at 40 kV/25 mA.

**Section S2. Antimicrobial screening**

Each compound was dissolved in DMSO, and solutions at a concentration of 1 mg/ml were prepared separately. Paper discs of Whatman filter paper, standard size (6 mm) were cut and sterilized in an autoclave. The paper discs soaked in the desired concentration of the complex solution were placed aseptically in the petri dishes containing nutrient agar media (agar 20g + beef extract 3g + peptone 5g) seeded with *Staphylococcus aureus, Bacillus subtilis, E. coli, Pseudomonas aeruginosa, Candida albicans, Proteus vulgaris* and *Aspergillus* fumigatus*.* The petri dishes were incubated at 36 ^°^C, and the inhibition zones were recorded after 24 h of incubation. Each treatment was replicated three times. The antibacterial activity of a common standard antibiotic, Gentamycin and antifungal Ketoconazole was also recorded using the same procedure as above at the same concentration and solvents.

**Section S3. Cytotoxic evaluation**

The cells were grown on RPMI-1640 medium supplemented with 10% inactivated fetal calf serum and 50µg/ml gentamycin. The cells were maintained at 37ºC in a humidified atmosphere with 5% CO_2_ and were subcultured two to three times a week.

For antitumor assays, the tumor cell lines were suspended in medium at a concentration 5x10^4^ cells/well in Corning® 96-well tissue culture plates, then incubated for 24 hr. The tested compounds were then added to 96-well plates (three replicates) to achieve ten concentrations for each compound. Six vehicle controls with media or 0.5 % DMSO were run for each 96 well plate as a control. After incubating for 48 h, the numbers of viable cells were determined by the MTT test. Briefly, the media was removed from the 96 well plate and replaced with 100 µl of fresh culture RPMI 1640 medium without phenol red then 10 µl of the 12 mM MTT stock solution (5 mg of MTT in 1 mL of PBS) to each well, including the untreated controls. The 96 well plates were then incubated at 37°C and 5% CO_2_ for 4 hours. An 85 µl aliquot of the media was removed from the wells, and 50 µl of DMSO was added to each well and mixed thoroughly with the pipette and incubated at 37°C for 10 min. Then, the optical density was measured at 590 nm with the microplate reader (SunRise, TECAN, Inc, USA) to determine the number of viable cells and the percentage of viability was calculated as [(ODt/ODc)]x100% where ODt is the mean optical density of wells treated with the tested sample and ODc is the mean optical density of untreated cells. The relation between surviving cells and drug concentration is plotted to get the survival curve of each tumor cell line after treatment with the specified compound. The 50% inhibitory concentration (IC_50_), the concentration required to cause toxic effects in 50% of intact cells, was estimated from graphic plots of the dose response curve for each conc. using Graphpad Prism software (San Diego, CA. USA).

**Section S4. Molecular docking simulation**

MOE-Dock 2014 is the program utilized for docking simulation. Using the builder button, the structures of ligands and complexes have been drawn. The energy of the six tested derivatives has been minimized through the utility the field of MMFF94x force in MOE-Dock 2014.09 program. The first step, all water molecules were removed via the molecular docking process. Then, addition of the lost hydrogen atoms was achieved through the suitable ionization-states of the selected protein and followed by running the “Docking” module in MOE program to carry out the molecular docking step. The top 30 diverse positions of London dG were obtained and lowered by MMFF94x within a solid receiver. GBVI/WSA dG recording function was used for scoring. The “Ligand 28 Interactions” tool was applied to solve the outputs by visualizing the Legend enzyme interactions within the complex active site and to display the results in the form of a graph, and to show the different interactions between the receptor and the ligand. Among the main interactions included are the H-bonds, hydrophobic, π–π and cation-π interactions.


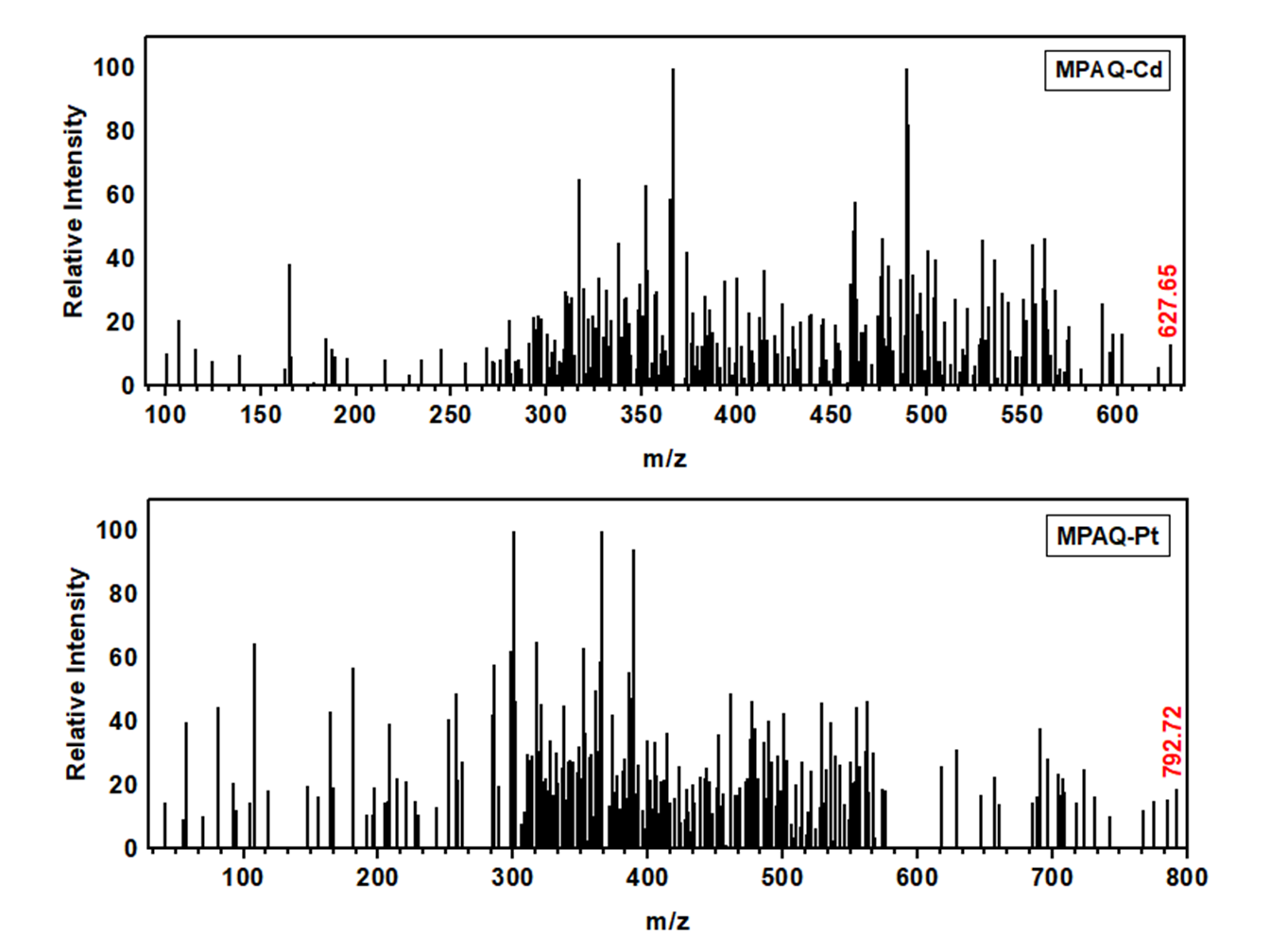


Fig. S1. Mass spectra of **MPAQ-Cd** and **MAPQ-Pt**


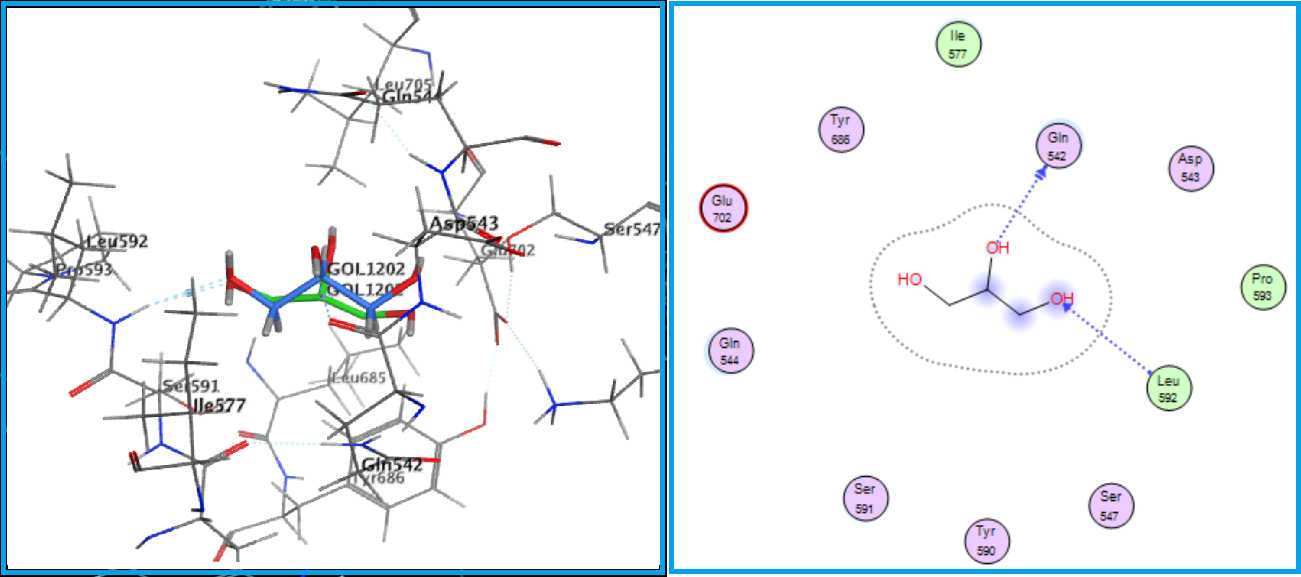


**Fig. S2.** (left) 3D snapshots of the validation of MOE showing the superimposition of the native co-crystallized ligand (blue) with GLY (green) poses i. (Right) 2D interactions of the co-crystal ligand (GLY) with 4fm9.


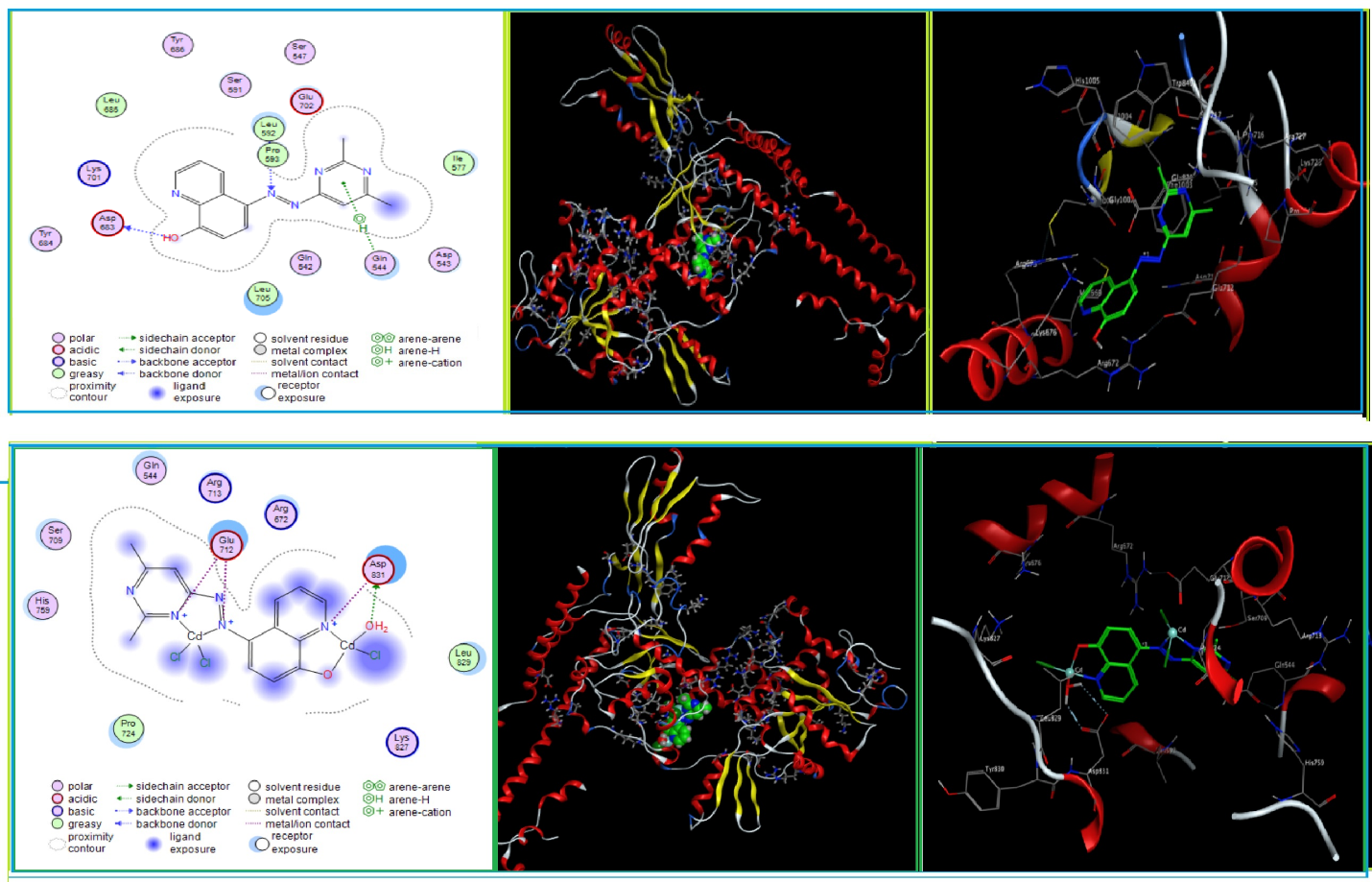


**Fig. S3.** 2D and 3D snaps of the binding modes of MPAQ and MPAQ-Cd into the active site of the protein with pdb code 4fm9


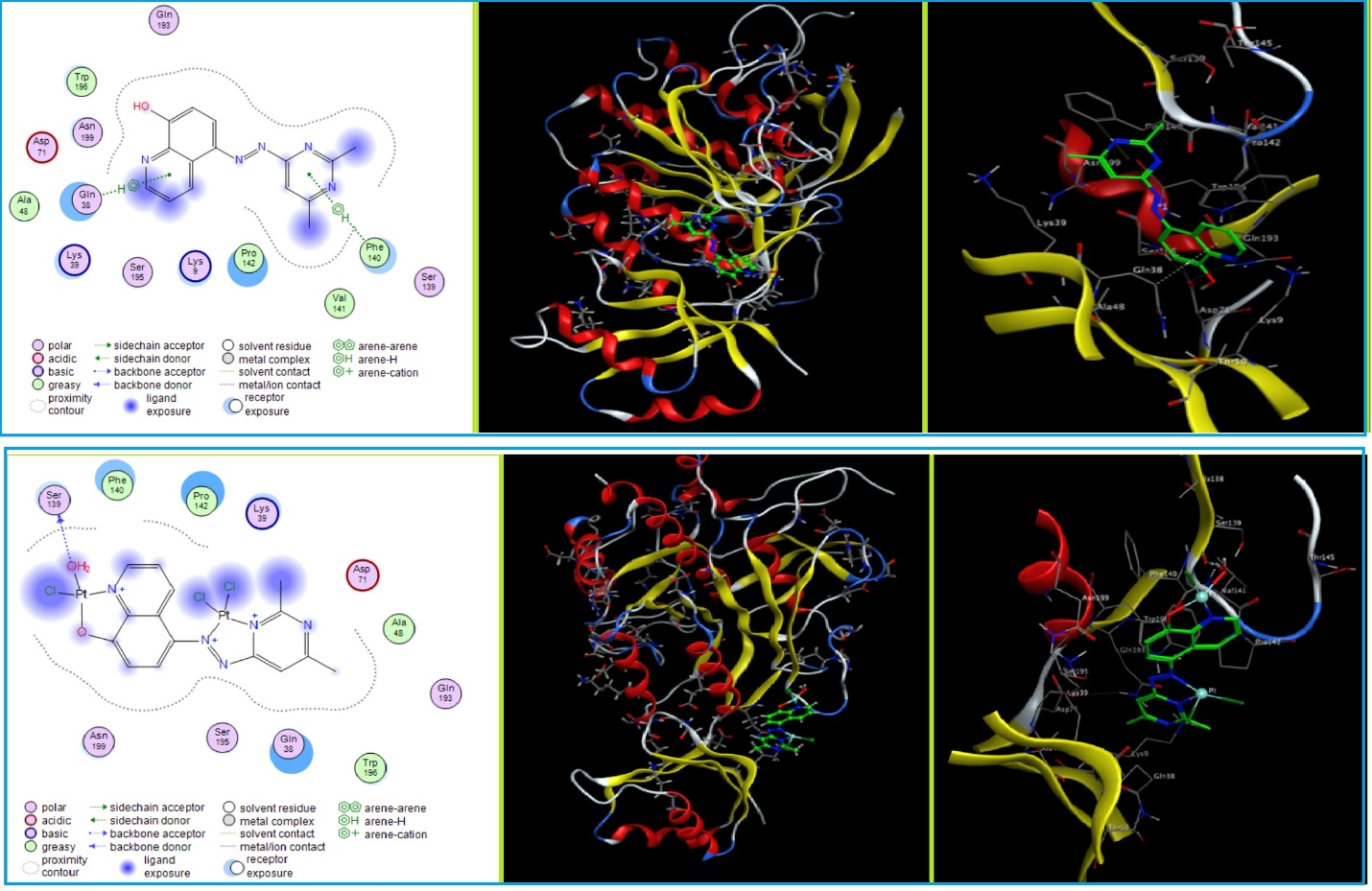


**Fig. S4.** 2D and 3D snaps of the binding modes of MPAQ and MPAQ-Pt into the active site of the protein with pdb code 3WHI

**
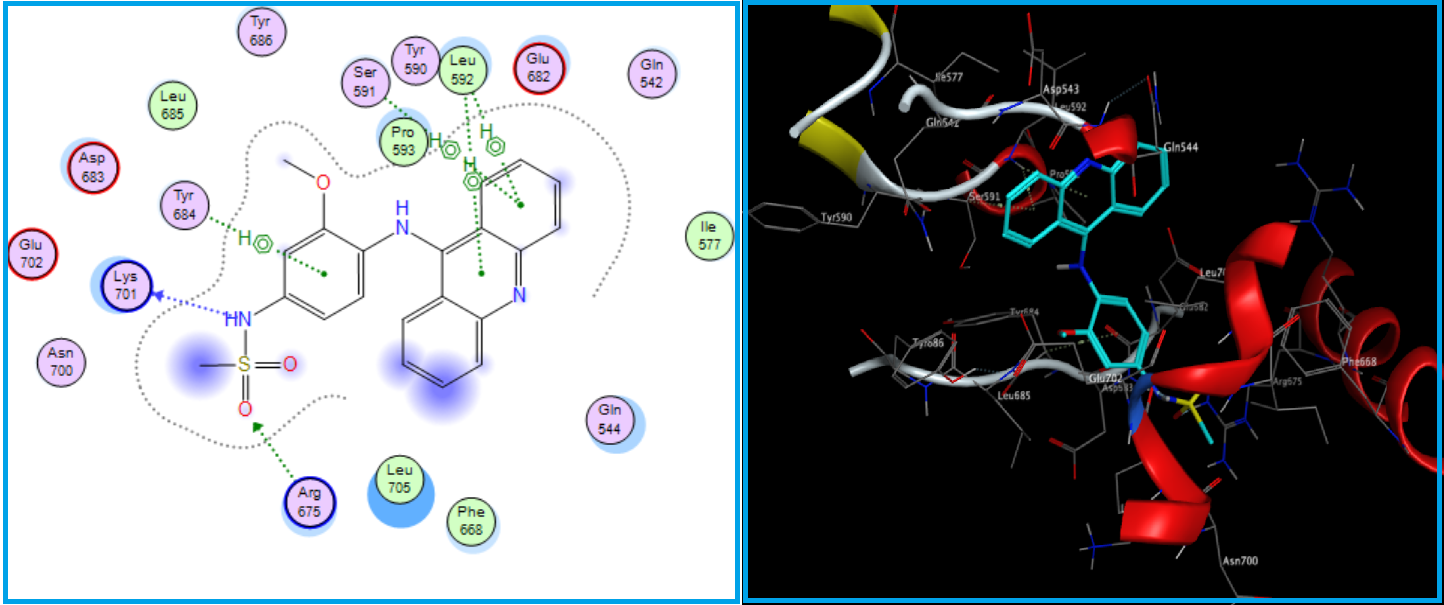
Fig. S5.** 2D and 3D snaps of the binding modes of the reference drug Amsacrine into the active site of the protein with pdb code 4fm9

**Table S1.**  Assignment for diagnostic important bands (cm^-1^) in IR spectra for **MPAQ** and its complexes

| **Comp.** | ν(OH) | ν(C=N)  Pyrimidine | ν(C=N)  quinoline | ν(N=N) | ν(C-O) | ν(M-O) | ν(M-N) |
| --- | --- | --- | --- | --- | --- | --- | --- |
| **MPAQ** | 3391 | 1651 | 1564 | 1452 | 1205 | - | - |
| **MPAQ-Ni** | 3493 | 1657 | 1578 | 1463 | 1233 | 504 | 440 |
| **MPAQ-Cd** | 3457 | 1606 | 1577 | 1460 | 1231 | 544 | 442 |
| **MPAQ-Pt** | 3402 | 1638 | 1576 | 1461 | 1220 | 585 | 421 |

**Table S2.** Selected bond lengths (Å), angles (º) and net charge (e) on atoms for the investigated ligand and its Pt-complex.

| **MPAQ** | | | | | | **MPAQ** | | | | | | |
| --- | --- | --- | --- | --- | --- | --- | --- | --- | --- | --- | --- | --- |
| Charges (e) | | Bond angles (º) | | Bond length (Å) | | Charges (e) | | | Bond angles (º) | | Bond length (Å) | |
| -0.398 | N_1_ | 114.258 | N_1_C_10_C_9_ | 1.344 | N_1_C_2_ | -0.412 | | N_1_ | 114.820 | N_1_C_10_C_9_ | 1.319 | N_1_C_2_ |
| -0.197 | C_2_ | 120.120 | C_2_N_1_C_10_ | 1.385 | N_1_C_10_ | 0.165 | | C_2_ | 118.330 | C_2_N_2_C_10_ | 1.354 | N_1_C_10_ |
| 0.334 | C_5_ | 119.302 | C_5_C_6_C_7_ | 1.450 | C_5_C_6_ | 0.096 | | C_5_ | 118.782 | C_5_C_6_C_7_ | 1.443 | C_5_C_6_ |
| 0.277 | C_6_ | 116.787 | C_7_C_6_N_12_ | 1.420 | C_6_C_7_ | -0.521 | | C_6_ | 112.395 | C_7_C_6_N_12_ | 1.394 | C_6_C_7_ |
| -0.398 | C_8_ | 117.036 | C_6_N_12_N_13_ | 1.405 | C_8_C_9_ | -0.386 | | C_8_ | 118.479 | C_6_N_12_N_13_ | 1.379 | C_8_C_9_ |
| 0.290 | C_9_ | 118.511 | C_8_C_9_C_10_ | 1.443 | C_9_C_10_ | 0.919 | | C_9_ | 120.069 | C_8_C_9_C_10_ | 1.436 | C_9_C_10_ |
| 0.072 | C_10_ | 117.900 | C_10_C_9_O_11_ | 1.338 | C_9_O_11_ | -0.066 | | C_10_ | 121.710 | C_8_C_9_O_11_ | 1.339 | C_9_O_11_ |
| -0.407 | O_11_ | 113.155 | N_12_N_13_C_14_ | 1.334 | N_12_N_13_ | -0.995 | | O_11_ | 118.220 | C_10_C_9_O_11_ | 1.258 | N_12_N_13_ |
| -0.373 | N_12_ | 119.152 | N_13_C_14_N_15_ | 1.386 | N_13_C_14_ | -0.615 | | N_12_ | 113.970 | N_12_N_13_C_14_ | 1.422 | N_13_C_14_ |
| -0.129 | N_13_ | 119.411 | N_13_C_14_C_19_ | 1.402 | N_12_C_6_ | -0.665 | | N_13_ | 120.831 | N_13_C_14_N_15_ | 1.398 | N_12_C_6_ |
| 0.311 | C_14_ | 81.456 | N_1_PtO_11_ | 1.387 | C_14_N_15_ | 0.900 | | C_14_ | 116.752 | N_13_C_14_C_19_ | 1.332 | C_14_N_15_ |
| -0.435 | N_15_ | 100.252 | N_1_PtO_39_ | 1.406 | C_14_ C_19_ | -0.667 | N_15_ | | 122.362 | N_15_C_14_C_19_ | 1.396 | C_14_ C_19_ |
| 0.168 | Pt_34_ | 98.017 | O_11_PtCl_38_ | 2.038 | PtN_1_ |  | | | | | | |
| 0.304 | Pt_37_ | 80.304 | Cl_38_PtO_39_ | 2.006 | PtO_11_ |  |  |  |  |  |  |  |
| -0.168 | Cl_35_ | 177.874 | N_1_PtCl_38_ | 2.411 | PtCl_38_ |  |  |  |  |  |  |  |
| -0.169 | Cl_36_ | 178.137 | O_11_PtO_39_ | 2.130 | PtO_39_ |  |  |  |  |  |  |  |
| -0.220 | Cl_38_ | 78.300 | N_12_PtN_15_ | 2.026 | PtN_12_ |  |  |  |  |  |  |  |
| -0.655 | O_39_ | 95.761 | N_12_PtCl_35_ | 2.069 | PtN_15_ |  |  |  |  |  |  |  |
|  | | 99.106 | N_15_PtCl_36_ | 2.400 | PtCl_35_ |  |  |  |  |  |  |  |
|  |  | 86.057 | Cl_35_PtCl_36_ | 2.391 | PtCl_36_ |  |  |  |  |  |  |  |
|  |  | 171.278 | N_12_PtCl_35_ |  | |  |  |  |  |  |  |  |
|  |  | 173.079 | N_15_PtCl_36_ |  |  |  |  |  |  |  |  |  |

**Table S3.** Selected bond lengths (Å), angles (º) and net charge (e) on atoms for Ni (II) and Cd(II) complexes.

| **MPAQ-Cd** | | | | | | **MPAQ-Ni** | | | | | | |
| --- | --- | --- | --- | --- | --- | --- | --- | --- | --- | --- | --- | --- |
| Charges (e) | | Bond angles (º) | | Bond length (Å) | | Charges (e) | | Bond angles (º) | | | Bond length (Å) | |
| -0.270 | N_1_ | 118.841 | N_1_C_10_C_9_ | 1.332 | N_1_C_2_ | -0.344 | N_1_ | 118.570 | | N_1_C_10_C_9_ | 1.474 | N_1_C_2_ |
| -0.259 | C_2_ | 124.287 | C_2_N_1_C_10_ | 1.340 | N_1_C_10_ | -0.379 | C_2_ | 111.748 | | C_2_N_1_C_10_ | 1.450 | N_1_C_10_ |
| 0.355 | C_5_ | 120.271 | C_5_C_6_C_7_ | 1.539 | C_5_C_6_ | 0.212 | C_5_ | 119.252 | | C_5_C_6_C_7_ | 1.355 | C_5_C_6_ |
| 0.154 | C_6_ | 119.891 | C_7_C_6_N_12_ | 1.421 | C_6_C_7_ | 0.256 | C_6_ | 120.371 | | C_7_C_6_N_12_ | 1.552 | C_6_C_7_ |
| -0.382 | C_8_ | 126.564 | C_6_N_12_N_13_ | 1.404 | C_8_C_9_ | -0.302 | C_8_ | 124.665 | | C_6_N_12_N_13_ | 1.540 | C_8_C_9_ |
| 0.226 | C_9_ | 118.280 | C_8_C_9_C_10_ | 1.598 | C_9_C_10_ | 0.278 | C_9_ | 118.817 | | C_8_C_9_C_10_ | 1.361 | C_9_C_10_ |
| -0.067 | C_10_ | 119.019 | C_10_C_9_O_11_ | 1.359 | C_9_O_11_ | 0.162 | C_10_ | 117.755 | | C_10_C_9_O_11_ | 1.455 | C_9_O_11_ |
| -0.519 | O_11_ | 117.176 | N_12_N_13_C_14_ | 1.326 | N_12_N_13_ | -0.517 | O_11_ | 113.083 | | N_12_N_13_C_14_ | 1.302 | N_12_N_13_ |
| -0.178 | N_12_ | 118.064 | N_13_C_14_N_15_ | 1.566 | N_13_C_14_ | -0.258 | N_12_ | 115.598 | | N_13_C_14_N_15_ | 1.511 | N_13_C_14_ |
| -0.154 | N_13_ | 123.595 | N_13_C_14_C_19_ | 1.470 | N_12_C_6_ | -0.130 | N_13_ | | 125.639. | N_13_C_14_C_19_ | 1.470 | N_12_C_6_ |
| 0.208 | C_14_ | 91.093 | N_1_CdO_11_ | 1.360 | C_14_N_15_ | 0.324 | C_14_ | | 113.671 | N_1_NiCl_23_ | 1.327 | C_14_N_15_ |
| -0.275 | N_15_ | 115.691 | N_1_CdO_27_ | 1.390 | C_14_ C_19_ | -0.388 | N_15_ | | 97.607 | N_1_NiO_11_ | 1.527 | C_14_ C_19_ |
| 0.638 | Cd_22_ | 112.949 | N_1_CdCl_26_ | 2.041 | CdN_1_ | 0.364 | Ni_22_ | | 113.963 | N_1_NiO_27_ | 1.891 | NiN_1_ |
| -0.463 | Cl_23_ | 111.750 | O_11_CdCl_26_ | 2.028 | CdO_11_ | -0.073 | Ni_24_ | | 114.672 | O_11_NiCl_23_ | 1.995 | NiO_11_ |
| -0.467 | Cl_24_ | 115.637 | O_11_CdO_27_ | 2.400 | CdCl_23_ | -0.327 | Cl_23_ | | 112.918 | O_11_NiO_27_ | 2.293 | NiCl_23_ |
| 0.900 | Cd_25_ | 108.910 | Cl_26_CdO_27_ | 2.038 | CdCl_24_ | -0.245 | Cl_25_ | | 104.408 | Cl_23_NiO_27_ | 1.969 | NiO_27_ |
| -0.420 | Cl_26_ | 109.422 | Cl_23_CdCl_24_ | 2.049 | CdN_12_ | -0.321 | Cl_26_ | | 102.242 | Cl_25_NiCl_26_ | 1.829 | NiN_12_ |
| -0.765 | O_27_ | 116.636 | N_12_CdCl_23_ | 2.323 | CdN_15_ | -0.670 | O_27_ | | 117.214 | N_12_NiCl_25_ | 1.812 | NiN_15_ |
|  | | 112.559 | N_12_CdCl_24_ | 2.400 | CdCl_26_ |  | | | 116.693 | N_15_NiCl_26_ | 2.140 | NiCl_25_ |
|  |  | 112.161 | N_15_CdCl_24_ | 2.070 | CdCl_27_ |  |  |  | 115.017 | Cl_26_NiN_12_ | 2.140 | NiCl_26_ |
|  |  | 117.038 | Cl_23_NiN_15_ |  | |  |  |  | 115.542 | Cl_26_NiN_15_ |  | |
|  |  | 87.674 | N_12_NiN_15_ |  |  |  |  |  | 90.915 | N_12_NiN_15_ |  |  |
